# Nano‐Enabled Systemic Delivery of STING Agonist by Engineered Silicasome for Potent Antitumor Immunotherapy

**DOI:** 10.1002/advs.202523722

**Published:** 2026-04-09

**Authors:** Wenjing Zhou, Yuting Li, Xudong Sun, Honghong Yang, Xiayu Shi, Lijia Yao, Jinhong Jiang, Erli Wang, Haoyang Wang, Yi Wei, Xiangsheng Liu

**Affiliations:** ^1^ School of Life Sciences Tianjin University Tianjin China; ^2^ Zhejiang Cancer Hospital The Key Laboratory of Zhejiang Province for Basic and Clinical Application of Functional Nucleic Acids Hangzhou Institute of Medicine (HIM) Chinese Academy of Sciences Hangzhou China; ^3^ Fuyang Institute Zhejiang Chinese Medical University Hangzhou China; ^4^ College of Pharmaceutical Science Zhejiang University of Technology Hangzhou China

**Keywords:** cancer immunotherapy, cyclic dinucleotide, silicasome nanocarrier, STING agonist, systemic delivery

## Abstract

Activation of the STING pathway represents a potent strategy for cancer immunotherapy. However, the instability and systemic toxicity of STING agonists, especially cyclic dinucleotides (CDNs), limit their use via local administration with poor efficacy against metastatic or inaccessible tumors. Here, we develop an engineered silicasome nanocarrier for systemic delivery of the CDN ADU‐S100 (ADU‐Sili). Comprising ADU‐S100‐loaded mesoporous silica nanoparticles (MSNPs) coated with lipid bilayer, ADU‐Sili markedly enhanced tumor accumulation and antitumor efficacy compared with free ADU‐S100 and conventional liposomes in vivo. The advantage of systemic delivery was further demonstrated in bilateral tumors: ADU‐Sili induced systemic antitumor immunity, suppressing both tumors, whereas free ADU‐S100 inhibited only injected tumors. Immune profiling revealed that ADU‐Sili promoted dendritic cell maturation in lymph nodes, expanded cytotoxic and memory CD8^+^ T cells in spleens, elevated intratumoral effector cytokines, and polarized tumor‐associated macrophages toward an M1 phenotype. Notably, combining ADU‐Sili with immune checkpoint blockade (ICB) synergistically enhanced antitumor efficacy as demonstrated in more clinically relevant orthotopic tumor models. In summary, silicasomes enable effective systemic CDN delivery, eliciting robust immune and antitumor responses and overcoming intratumoral delivery limitations in STING‐based cancer immunotherapy.

## Graphical abstract

Engineered mesoporous silica nanoparticles (Silicasomes) enable systemic delivery of the STING agonist ADU‐S100, overcoming the instability and toxicity that limit cyclic dinucleotides to local administration. By enhancing tumor accumulation, activating systemic antitumor immunity, and remodeling the tumor immune microenvironment, these nanoparticles effectively suppress multifocal tumor progression, offering a potent platform for cancer immunotherapy.

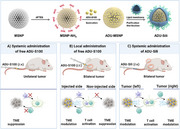

## 1 Introduction

Despite remarkable progress in cancer immunotherapy over the past decade, particularly with immune checkpoint blockade (ICB) strategies such as anti‐PD‐1/PD‐L1 and anti‐CTLA‐4 antibodies, most patients with solid tumors still fail to achieve durable responses [1, 2, 3, 4]. Improved prognostic outcomes in a wide range of malignancies are closely linked to abundant infiltration of tumor‐infiltrating lymphocytes (TILs), highlighting their pivotal role as both indicators of host immune competence and active mediators of antitumor responses [5, 6, 7]. “Cold” tumors, characterized by insufficient cytotoxic T lymphocyte (CTL) infiltration and an immunosuppressive tumor microenvironment (TME), present formidable therapeutic challenges [8, 9]. Even in initially responsive tumors, barriers within the TME, impaired antigen presentation, or weak type I interferon (IFN‐I) signaling often compromise therapeutic efficacy, and combination regimens frequently yield suboptimal outcomes [10, 11, 12].

IFN‐I has emerged as a key regulator that integrates innate and adaptive immunity, a process crucial for effective tumor eradication [13]. Accumulating preclinical evidence highlights the pivotal role of IFN‐I in effective antitumor immunity [14, 15]. Conversely, defective or suppressed IFN‐I signaling is associated with poor T cell infiltration, reduced responsiveness to ICB, and an unfavorable prognosis across multiple cancer types. The stimulator of interferon genes (STING), an adaptor protein localized to the endoplasmic reticulum membrane, detects cytosolic DNA and induces IFN‐I, thereby activating the innate immune system. STING pathway activation has thus emerged as one of the most promising strategies to elicit robust antitumor immunity [16]. This cascade drives the maturation of dendritic cells (DCs), cross‐presentation of tumor antigens, and priming of CD8^+^ CTLs [17, 18, 19]. Preclinical studies have demonstrated that pharmacological activation of STING converts cold tumors into highly immunoresponsive phenotypes by eliciting potent innate and adaptive immune responses [20, 21].

STING agonists are promising for cancer immunotherapy, with their translational potential being actively evaluated in preclinical and clinical studies. Cyclic dinucleotides (CDNs) represent the most classical STING agonists that directly activate the STING pathway and exhibit potential antitumor activity in multiple preclinical models [22, 23]. Among them, cyclic GMP‐AMP (cGAMP) is a natural CDN synthesized by cGAMP synthase (cGAS) upon recognition of cytoplasmic DNA, including viral, bacterial, or host‐derived DNA. Intracellular delivery of CDNs is required for STING binding and subsequent pathway activation. However, their clinical translation via systemic administration is hindered by their hydrophilicity and negative charge, which impede cellular uptake, rapid enzymatic degradation, and short circulation [24, 25]. For example, CDN‐based STING agonists such as MK‐1454 and MIW815 (ADU‐S100) in clinical studies often require direct intratumoral injection, which fails to induce systemic immunity and restricts broader clinical application [23]. In contrast, systemic administration of STING agonists can induce inflammatory cytokines in tumors, offering an appealing alternative to overcome these challenges [26, 27].

To overcome these limitations, nanoparticle‐based delivery platforms have been developed to enable controlled and effective systemic activation of the STING pathway. Nanoparticle formulations can encapsulate CDNs, protecting them from enzymatic degradation and enhancing cytosolic delivery. Additionally, nanoparticles enhance pharmacokinetics (PK) by prolonging circulation half‐life, promoting tumor accumulation, and reducing systemic toxicity [24, 28]. Consequently, employing nanoparticles to deliver STING agonists is emerging as a pivotal strategy to strengthen antitumor efficacy. Recent studies have demonstrated that nanocarrier‐based delivery systems can effectively overcome the poor cellular uptake and limited cytosolic transport of free STING agonists. For instance, biodegradable mesoporous silica nanoparticles have been engineered to enhance cellular uptake of STING agonists, resulting in efficient immune activation and potent antitumor responses following intratumoral injection in melanoma models [29]. In another approach, Mn^2^^+^‐crosslinked alginate gel nanocarriers have been developed to co‐deliver STING agonists and immune checkpoint inhibitors via intratumoral administration, promoting DC maturation and robust cytotoxic T‐cell activation [30]. Moreover, intratumoral injection of reduction‐responsive biodegradable chimeric polymersomes enables superior tumor retention and cytosolic delivery of synthetic CDNs such as ADU‐S100, amplifying STING pathway activation and synergizing with radiotherapy to achieve durable antitumor immunity [31]. Despite these advances, most nanocarrier systems still rely on intratumoral administration, which significantly limits their clinical applicability and translational potential.

In this study, we developed a systemically deliverable nanoparticle encapsulating STING agonist ADU‐S100 (ADU‐Sili) based on our previously established silicasome platform, composed of mesoporous silica nanoparticles (MSNPs) coated with a lipid bilayer. This nanocarrier already possesses tumor‐targeting capability, favorable biosafety, and scalable production, providing a robust foundation for systemic delivery [32, 33]. In vivo biodistribution studies demonstrated that the silicasome‐encapsulated STING agonist preferentially accumulated within the tumor microenvironment, highlighting the enhanced tumor‐targeting capability and improved pharmacokinetic profile conferred by the delivery system. In addition, a brief comparison with liposomes lacking the MSNP core showed that silicasomes achieved higher STING agonist loading and prolonged STING agonist circulation. We then evaluated the therapeutic efficacy of ADU‐Sili in multiple solid tumor models, including subcutaneous, bilateral, and orthotopic colorectal models, which are considered ideal for assessing STING agonist activity. In subcutaneous colorectal tumor models, systemic administration of the ADU‐Sili significantly enhanced antitumor efficacy compared with free ADU‐S100. The therapeutic advantage was even more pronounced in bilateral tumor models, where local administration of free ADU‐S100 suppressed growth only in the treated tumors, with no effect on the contralateral lesions. In contrast, systemic treatment with ADU‐Sili induced potent systemic antitumor immunity, effectively suppressing tumor progression at both sites. Furthermore, ADU‐Sili exhibited substantial antitumor activity in an orthotopic colorectal tumor model, which more closely mimics clinical conditions. Notably, the combination of ADU‐Sili with an anti‐PD‐1 (α‐PD‐1) antibody produced synergistic antitumor effects, resulting in greater tumor regression than either monotherapy alone. Notably, despite the intrinsic resistance of colorectal cancer to ICB therapy [34], the combination of ADU‐Sili with an α‐PD‐1 antibody elicited synergistic antitumor efficacy, resulting in significant tumor regression compared to either monotherapy.

**SCHEME 1:**
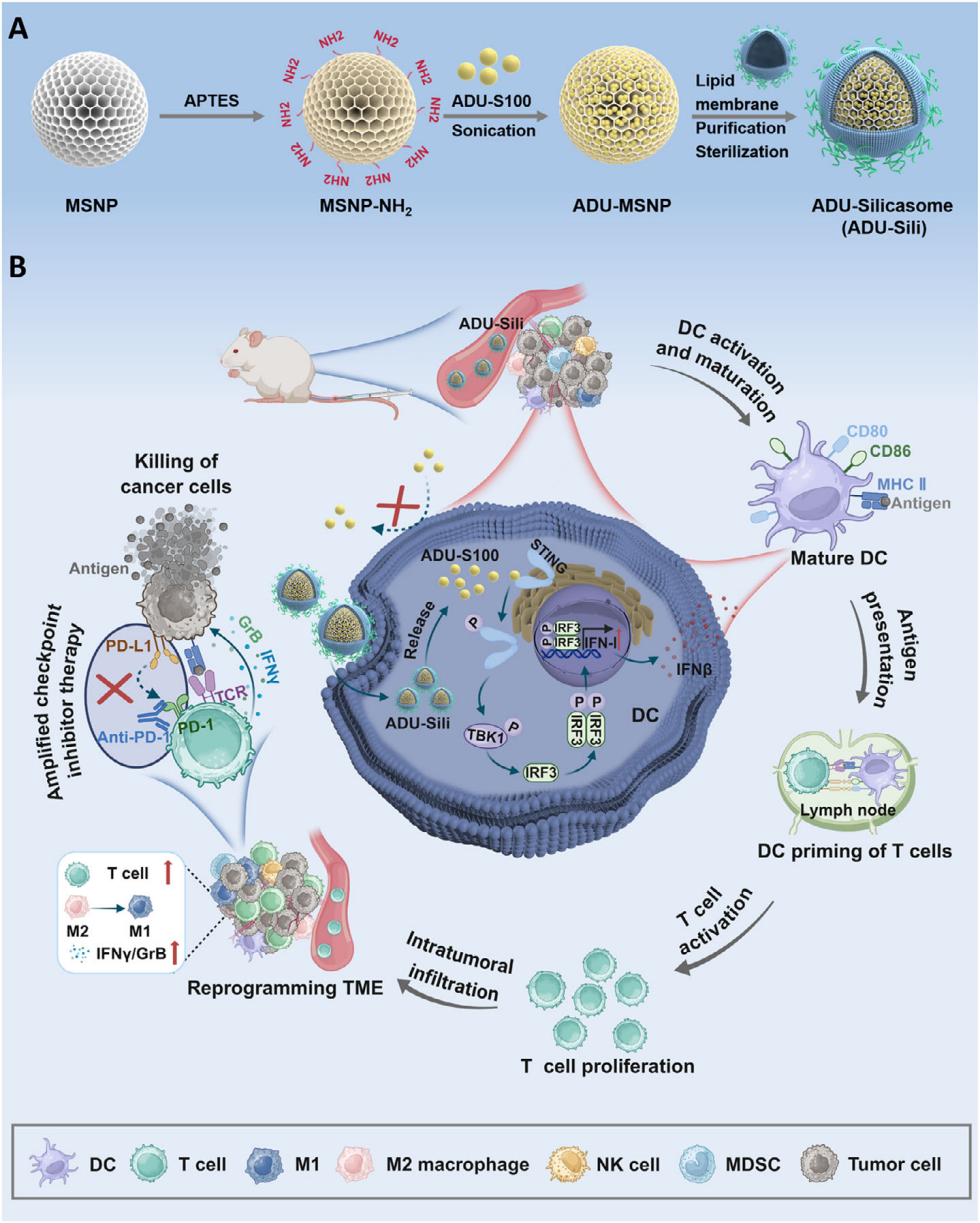
Schematic illustration of systemic delivery of ADU‐S100‐loaded silicasomes (ADU‐Sili) potentiates antitumor immunotherapy. (A) Rational design of ADU‐Sili for systemic delivery. (B) Mechanistic illustration of ADU‐Sili‐mediated potent antitumor immunity following systemic administration. (A) was created using Cinema 4D, and (B) was created using BioRender. Wenjing, Z. (2026) https://BioRender.com/ig3z96x.

Mechanistic investigations revealed that ADU‐Sili reshaped systemic and intratumoral immune landscapes. In tumor‐draining lymph nodes (TDLNs), treatment with ADU‐Sili elevated the proportion of mature DCs, reaching approximately 1.7‐fold higher than free ADU‐S100, indicating efficient antigen presentation. In the spleen, both CD8^+^ T cells and memory T cells were expanded, reflecting durable systemic immune activation. Within the TME, ADU‐Sili administration enhanced CD8^+^ CTL infiltration, accompanied by increased secretion of cytotoxic effector molecules, including granzyme B (GrB), interferon‐γ (IFN‐γ), and tumor necrosis factor‐α (TNF‐α). Furthermore, ADU‐Sili effectively reprogrammed tumor‐associated macrophages (TAMs) from an immunosuppressive M2 phenotype to a proinflammatory M1 state, thereby alleviating immunosuppression within the TME (Scheme 1).

**SCHEME 2:**
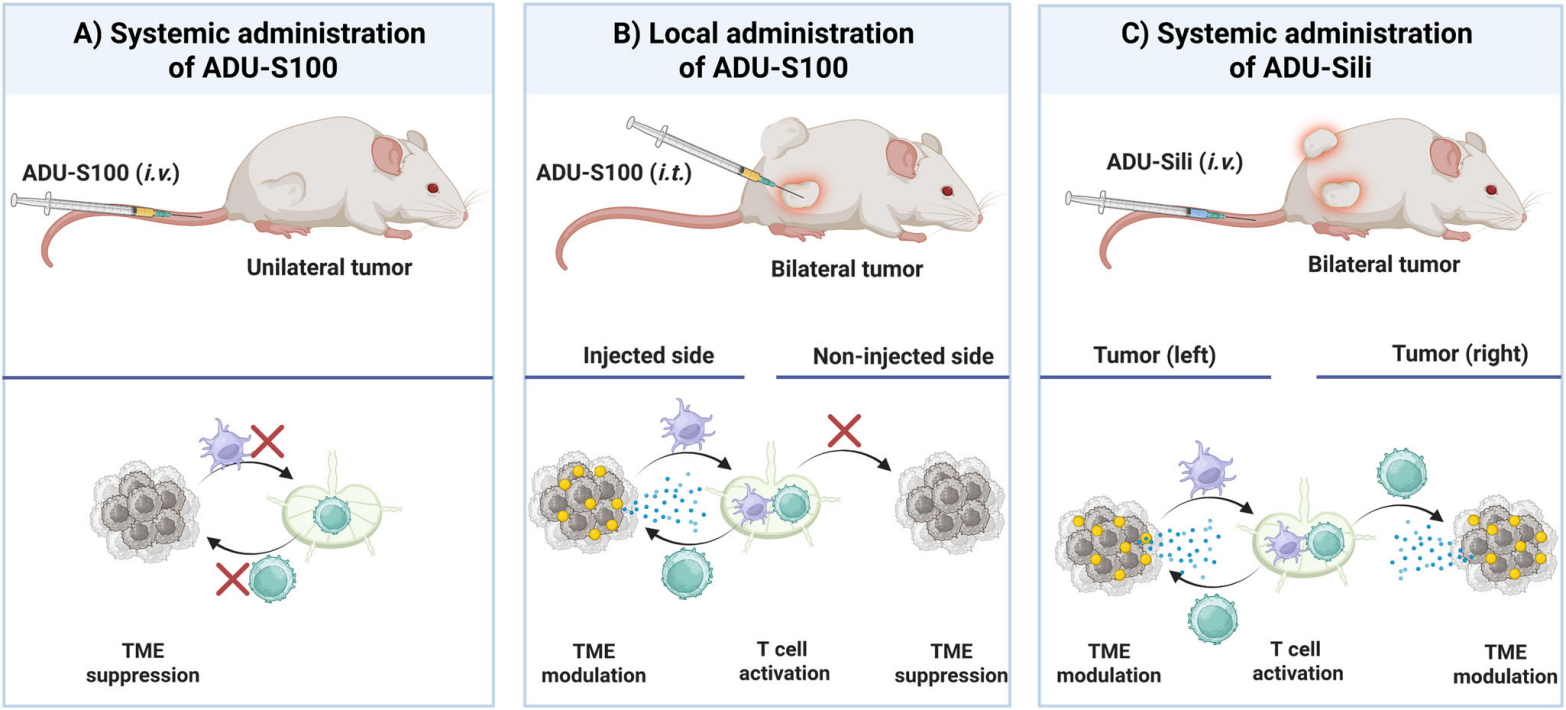
Systemic administration of ADU‐Sili activates systemic immunity and inhibits the progression of multifocal tumors, whereas ADU‐S100 exhibits limited systemic efficacy. Intratumoral ADU‐S100 induces a local immune response that suppresses the injected tumor but fails to inhibit distal tumors. Scheme 2 was created using BioRender. Wenjing, Z. (2026) https://BioRender.com/liotj9v.

Collectively, our findings demonstrate that systemic nanoparticle delivery of STING agonists not only improves biodistribution and immune activation but also overcomes the spatial limitations of intratumoral injection (Scheme 2). This study underscores the necessity for systemic administration of STING agonists and highlights the advantages of nanoparticle formulations for durable, potent antitumor immunity with minimized systemic toxicity. These findings highlight the promise of translating nanoparticle‐based STING agonist therapies into clinical practice, either as monotherapy or in combination with ICB.

## 2 Results

### 2.1 Loading of ADU‐S100 in Engineered MSNPs

**FIGURE 1:**
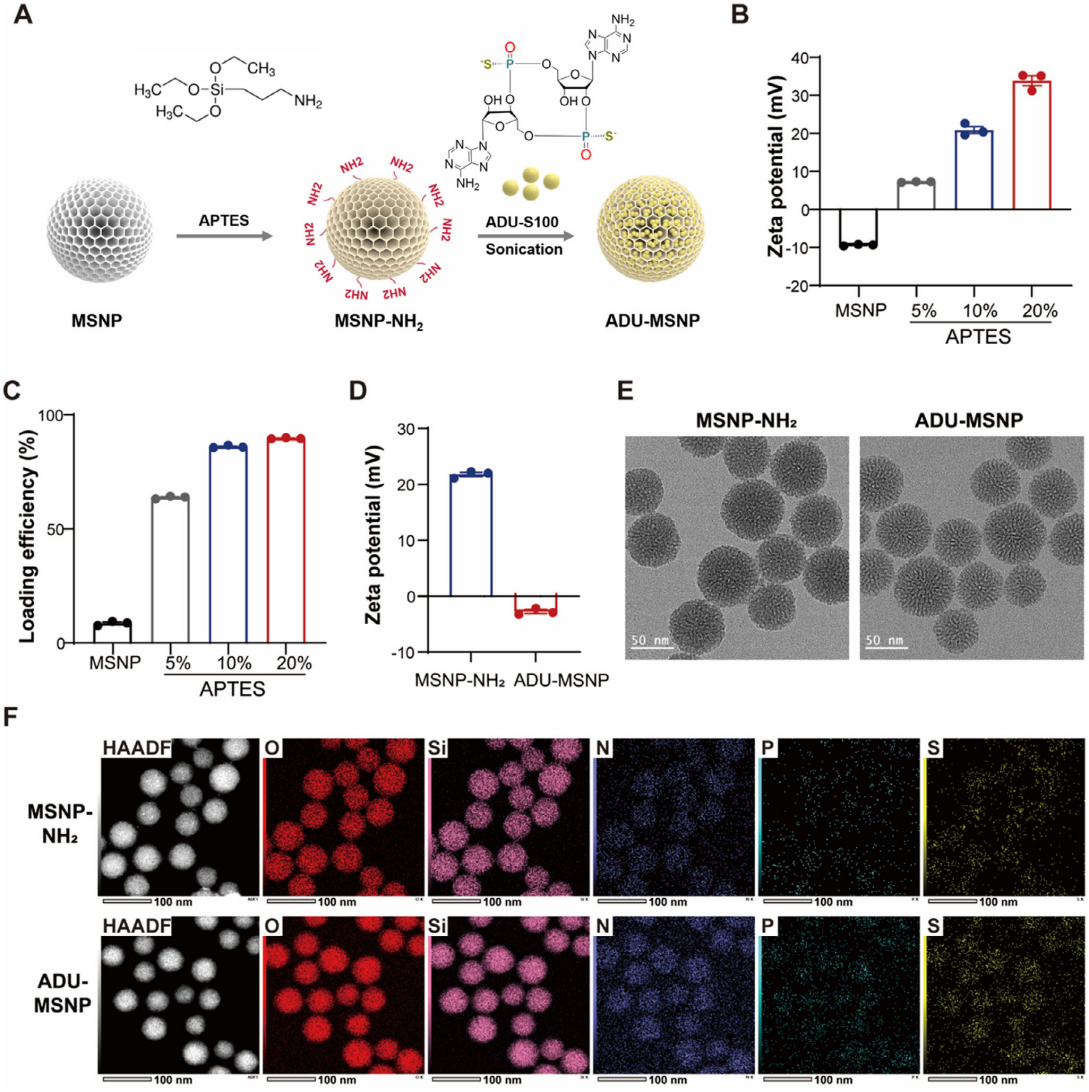
Preparation and characterization of ADU‐S100‐loaded MSNPs. (A) Schematic illustration of the loading of ADU‐S100 in engineered MSNPs. (B) Zeta potential of MSNPs functionalized with different ratios of APTES. (C) Loading efficiency of MSNPs varying with APTES modification. (D) Zeta potential of MSNPs before and after ADU‐S100 loading. (E) Representative TEM images of MSNP‐NH_2_ and ADU‐S100‐loaded MSNPs. Scale bar = 50 nm. (F) EDS elemental maps for O (red), Si (magenta), N (blue), P (green), and S (yellow) in the MSNP‐NH_2_ and ADU‐MSNPs. Scale bar = 100 nm. Data represent mean ± SEM.

The ADU‐S100 is synthesized according to the literature (Figure S1) [35], and its chemical structure was further validated by ^1^H and ^31^P NMR spectroscopy and mass spectrometry (Figures S2–S4). The bare MSNPs were synthesized as previously described [36]. To optimize the loading of negatively charged cyclic dinucleotide ADU‐S100, MSNPs were amine‐functionalized (MSNP‐NH_2_) by surface modification with 3‐aminopropyltriethoxysilane (APTES), imparting a positive charge, enabling the efficient ADU‐S100 loading in MSNP‐NH_2_ via electrostatic adsorption (Figure 1A). Amine modification progressively increased the zeta potential of bare MSNPs from −10 mV to approximately +5, +20, and +35 mV with increasing APTES ratio of 5%, 10%, and 20% (APTES volume/MSNP mass, µL/mg), respectively (Figure 1B). This observation indicates successful amine functionalization, leading to a positively charged surface. To optimize ADU‐S100 loading conditions, we assessed loading efficiency at varying APTES density (Figure 1C). Efficiency increased with APTES density, plateauing at 10%, which was selected for further experiments, and loading efficiency reached 86%. Upon loading with ADU‐S100, the zeta potential shifted to nearly neutral, confirming successful payload incorporation and surface charge neutralization (Figure 1D). The resulting ADU‐MSNPs had an approximate average diameter of 75 nm, with no discernible difference in appearance compared to MSNPs‐NH_2_, as visualized by transmission electron microscopy (TEM) (Figure 1E). Since the TEM images alone cannot provide information on the loading state of ADU‐S100, elemental mapping was performed by energy‐dispersive X‐ray spectroscopy (EDS) combined with field‐emission transmission electron microscopy (FE‐TEM) (Figure 1F). Following ADU‐S100 loading, a marked increase in featured N, P, and S signals confirmed its successful incorporation into MSNP‐NH_2_.

### 2.2 ADU‐MSNPs Amplify STING Activation and Tumor Suppression via Intratumoral Injection

**FIGURE 2:**
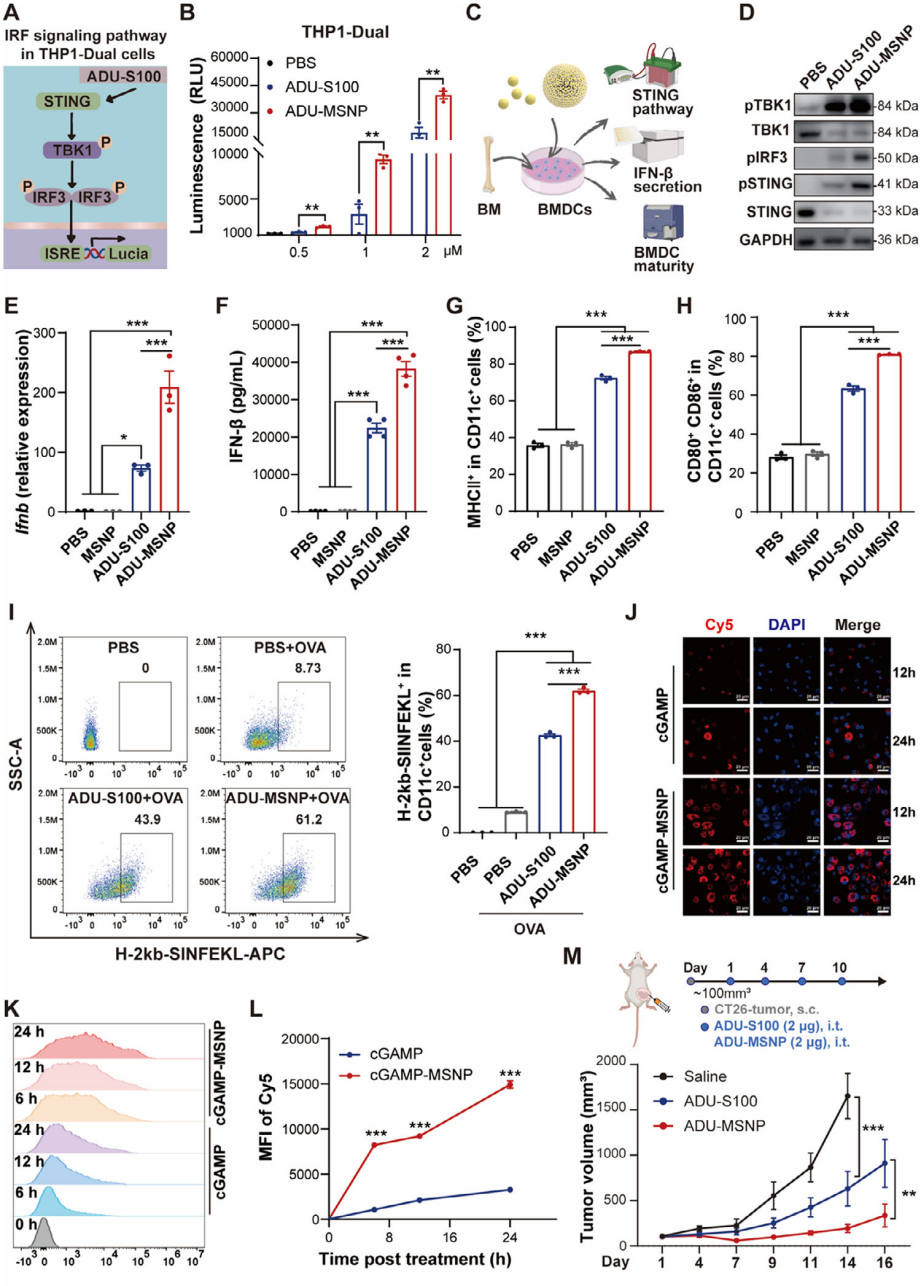
ADU‐MSNPs amplify STING activation and tumor suppression. (A) Schematic illustration of STING activation in IRF‐Luciferase reporter THP1‐Dual cell line. Luminescence intensity is proportional to the IFN‐I. (B) Dose‐response in THP1‐Dual cells after 24 h treatment with free ADU‐S100 and ADU‐MSNPs; RLU, relative light units (n = 3). (C) Experimental schematic for STING activation assays in BMDCs. (D) Representative immunoblots of the STING‐IFN‐I pathway in BMDCs treated for 6 h with 1 µM ADU‐S100 or ADU‐MSNPs. (E,F) Quantification of *Ifnb* mRNA by qRT‐PCR (E) and IFN‐β secretion by ELISA (F) in BMDCs after 24 h exposure to ADU‐S100 and ADU‐MSNPs (n = 3). (G,H) Flow cytometry analysis of BMDC maturation markers following 24 h incubation of ADU‐S100 and ADU‐MSNPs: major histocompatibility complex II (MHC II) (G), and combined CD80/CD86 expression (H) (n = 3). (I) Representative flow cytometry plots and quantification of ovalbumin (OVA, SIINFEKL)‐presenting BMDCs induced by ADU‐MSNPs. BMDCs were treated with ADU‐MSNPs for 24 h and subsequently pulsed with 1 µg/mL OVA_257–264_ peptide for 2 h (n = 3). (J–L) cGAMP‐MSNPs increased cellular internalization of cGAMP. BMDCs were treated with free cGAMP‐Cy5 or cGAMP‐Cy5‐MSNPs for 6, 12, or 24 h with 1 µM cGAMP, then analyzed by confocal microscopy (J) and flow cytometry (K,L) (n = 3). Scale bar = 20 µm. (M) In vivo treatment schedule for evaluation of ADU‐MSNPs therapeutic efficacy. Tumor growth curves monitored over time (n = 4). Data represent mean ± SEM. Statistical analysis was performed by two‐tailed Student's *t*‐test (B), or one‐way ANOVA with Tukey's multiple comparisons test (E–I), or two‐way ANOVA with Tukey's multiple comparisons test (L‐N). *p < 0.05, **p < 0.01, ***p < 0.001.

Given the pivotal role of the cGAS‐STING‐type I interferon (IFN‐I) pathway in antitumor immunity [37], we first evaluated the capacity of ADU‐MSNPs to stimulate IFN‐I production in THP1‐Dual reporter cells. In this assay system, IFN‐I was quantified by interferon‐stimulated response element (ISRE)‐driven luciferase activity (Figure 2A) [38]. THP1‐Dual cells were treated with PBS, ADU‐S100, or ADU‐MSNPs at concentrations ranging from 0.5 to 2 µM, and luciferase activity was assessed to evaluate IFN‐I signaling. ADU‐MSNPs elicited a dose‐dependent enhancement of luciferase activity, which consistently surpassed that induced by free ADU‐S100 at both 24 h and 48 h, thereby demonstrating a more robust activation of the cGAS‐STING‐IFN‐I pathway (Figure 2B, Figure S5).

To further assess the immunomodulatory effects of ADU‐MSNPs, we initially evaluated STING activation and maturation in primary bone marrow‐derived dendritic cells (BMDCs) (Figure 2C). Optimal IFN‐I gene transcription is critically dependent on interferon regulatory factor 3 (IRF3), a central transcription factor in innate immune signaling [39]. Accordingly, we investigated STING‐IRF3 axis activation by western blot. The results demonstrated that ADU‐MSNPs significantly enhanced the protein expression levels of phosphorylated STING, IRF3, and TANK‐binding kinase 1 (TBK1) compared to free ADU‐S100, indicating a robust activation of the STING signaling pathway (Figure 2D, Figure S6). Following treatment with ADU‐MSNPs, the effector cytokine interferon beta (IFN‐β) exhibited maximal induction at both the transcriptional (200‐fold than PBS) and translational levels (560‐fold than PBS), suggesting a potent immune‐stimulatory effect (Figure 2E,F). Subsequently, BMDC activation following ADU‐MSNPs treatment was quantified by flow cytometry. ADU‐S100 significantly upregulated surface co‐stimulatory molecules (MHC II, CD80, CD86) compared to PBS, while ADU‐MSNPs induced even higher expression, suggesting enhanced immunostimulatory activity (Figure 2G,H, Figure S7). In addition, control experiments confirmed that the APTES amination modification did not induce significant BMDC maturation, thereby excluding its contribution to the observed effects (Figure S8). To evaluate the antigen‐presenting capability, Ovalbumin (OVA) peptide (OVA_257–264_, SIINFEKL) was used as a model antigen, and the efficiency of antigen presentation was quantified by the expression of the H‐2K^b^‐SIINFEKL complex. Compared to the PBS group, OVA_257–264_ alone and ADU‐S100 plus OVA_257–264_ induced increased H‐2K^b^‐SIINFEKL expression, while ADU‐MSNPs plus OVA_257–264_‐treated BMDCs exhibited a sixfold higher expression than OVA_257–264_ alone (Figure 2I, Figure S9). To elucidate the mechanism of enhanced activation of BMDCs by ADU‐MSNPs, we used fluorophore‐labelled CDN, cGAMP‐Cy5, as a mimic to monitor cellular internalization of STING agonist. Free cGAMP‐Cy5 showed poor internalization, whereas cGAMP‐MSNPs resulted in significantly stronger intracellular fluorescence in a time‐dependent manner, indicating efficient MSNP‐mediated delivery (Figure 2J–L).

Given the robust immune activation induced by ADU‐MSNPs in vitro, we next investigated the therapeutic potential in vivo. A subcutaneous tumor model was established by inoculating BALB/c mice with CT26 colon cancer cells. When tumor volumes reached approximately 100 mm^3^, intratumoral (i.t.) injections of either ADU‐S100 or ADU‐MSNPs were administered. The results demonstrated that both ADU‐S100 and ADU‐MSNPs exhibited antitumor activity, with ADU‐MSNPs showing superior tumor growth inhibition and extended survival in tumor‐bearing mice (Figure 2M, Figure S10A–C). Notably, no significant body weight loss was observed in the treated mice (Figure S10D), indicating that the formulation is well tolerated.

### 2.3 Systemic Administration Facilitates Preferential Tumor Accumulation of STING Agonists

**FIGURE 3:**
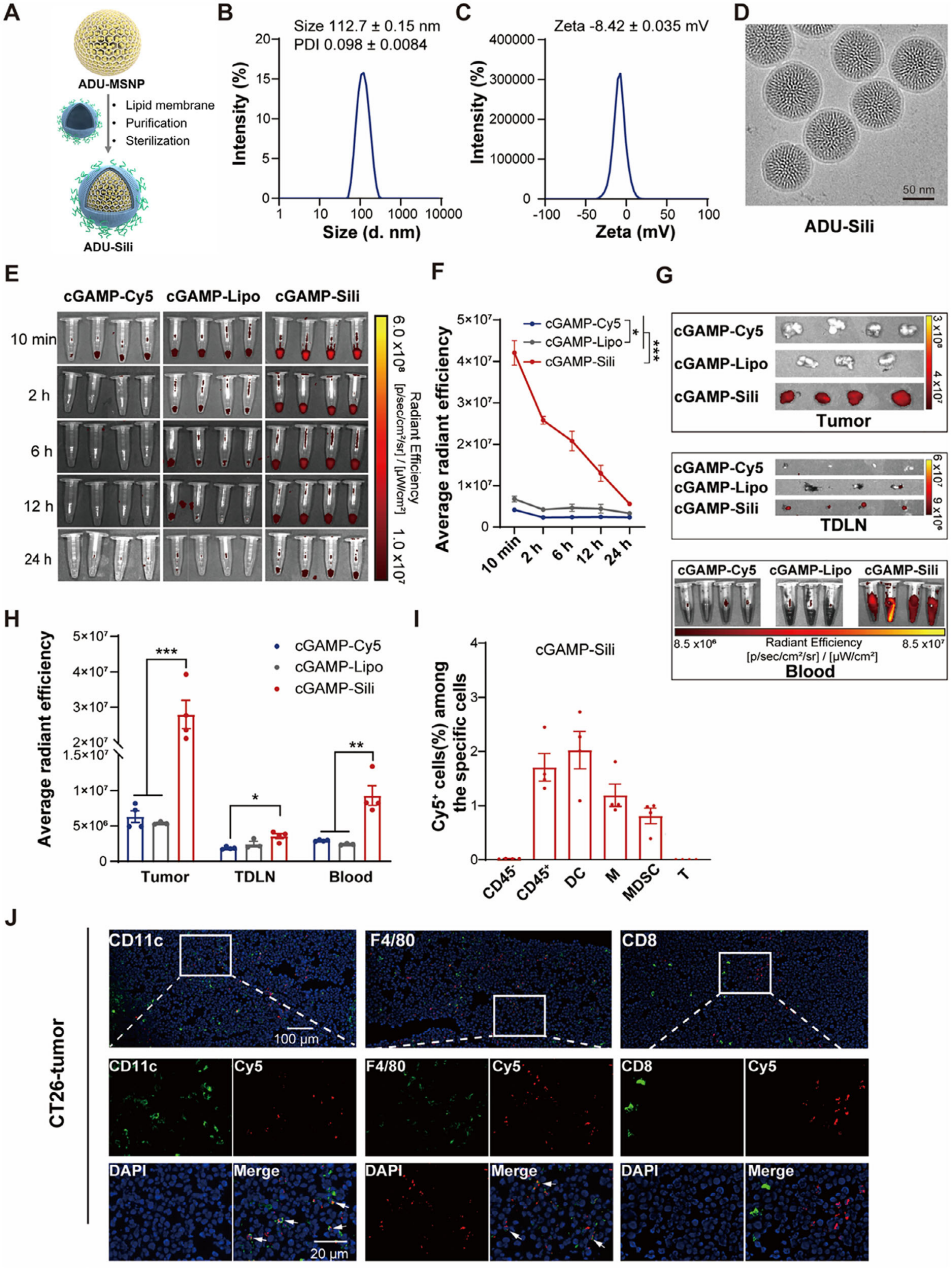
Systemic delivery by silicasomes facilitates preferential tumor accumulation of STING agonists. (A) Schematic illustration of the preparation of ADU‐Sili nanoparticle. (B,C) Hydrodynamic diameter (B) and zeta potential (C) of ADU‐Sili (n = 3). D) Representative Cryo‐TEM image of ADU‐Sili. (E,F) The circulation of CDNs loaded in silicasomes compared with the free drug or loaded in conventional liposomes using Cy5‐labeled cGAMP. IVIS images of cGAMP‐Cy5 signal in serum and quantification of average radiant efficiency (n = 4). (G,H) The in vivo biodistribution of cGAMP‐Cy5, cGAMP‐Lipo, and cGAMP‐Sili was evaluated in CT26 tumor‐bearing mice following intravenous administration. Biodistribution of cGAMP‐Cy5 in tumors, TDLNs, and blood (G), with quantification of average radiant efficiency across major organs and tumors (H) (n = 3–4). (I) Flow cytometry analysis of cGAMP‐Cy5 uptake among immune cell populations within the TME (n = 4). (J) Representative immunofluorescence images showing the intratumoral localization of cGAMP‐Sili. Red: cGAMP‐Sili; green, DCs staining with anti‐CD11c antibody, macrophages staining with anti‐F4/80 antibody, T cells staining with anti‐CD8 antibody; blue, nuclear stained with DAPI. Scale bar = 20 µm. Data represent mean ± SEM. Statistical analysis was performed by one‐way ANOVA with Tukey's multiple comparisons test (H), or two‐way ANOVA with Tukey's multiple comparisons test (F). *p < 0.05, **p < 0.01, ***p < 0.001.

Given their rapid degradation, most STING agonists in clinical trials are administered intratumorally. However, this approach is ineffective against inaccessible or metastatic tumors, highlighting the critical need for systemically deliverable, tumor‐targeted therapeutics. Although local injection of ADU‐MSNPs has exhibited potent antitumor activity, it is well‐known that bare MSNPs are unsuitable for intravenous administration due to their poor colloidal stability and rapid nonspecific clearance by the reticuloendothelial system (RES). To overcome these limitations, a lipid bilayer was subsequently introduced, which conferred enhanced colloidal stability and prolonged systemic circulation, as extensively demonstrated in previous studies (Figure 3A) [32, 36, 40, 41]. The lipid bilayer was composed of DSPC, cholesterol, and DSPE‐PEG2K at a molar ratio of 3:2:0.15. The lipid‐coated nanoparticles were designated as ADU‐Silicasomes (ADU‐Sili). The resulting particles exhibited an average diameter of 110 nm with a PDI below 0.15, indicating a uniform size distribution, and the zeta potential stabilized at −8 mV (Figure 3B,C). Cryo‐transmission electron microscopy (Cryo‐TEM) images confirmed a uniform lipid coating on the surface of the MSNPs (Figure 3D).

To evaluate the PK of STING‐activating silicasome nanoparticles, mice were intravenously administered free cGAMP‐Cy5, cGAMP‐Sili (cGAMP‐Cy5‐loaded silicasomes), or cGAMP‐Lipo (cGAMP‐Cy5‐loaded liposomes) lacking the MSNP core for comparison. The preparation and characterization of CDN‐Lipo are shown in Figure S11. Notably, ADU‐Lipo showed much lower loading efficiency than ADU‐Sili, which may be attributable to passive loading of hydrophilic CDNs that lack specific interactions with lipid membranes. Additionally, DiR dyes were incorporated into the lipid membrane to enable nanoparticle tracking. Serum samples collected at designated time points were analyzed by in vivo imaging to quantify Cy5 fluorescence. Compared to free cGAMP‐Cy5 and cGAMP‐Lipo, cGAMP‐Sili exhibited significantly higher fluorescence intensity, indicating prolonged systemic circulation of STING agonist (Figure 3E). Quantitative analysis of radiant efficiency showed that silicasome encapsulation markedly extended the systemic retention of cGAMP, with detectable signals up to 24 h. In contrast, Cy5 signals of free cGAMP and cGAMP‐Lipo declined rapidly within 2 and 6 h, respectively (Figure 3F). The circulation profile of silicasomes, as reflected by the DiR signal, closely mirrored the PK of loaded cGAMP (Figure S12A). cGAMP‐Lipo exhibited a similar DiR profile, reflecting the comparable nanoparticle surface (Figure S12B). Surprisingly, while the liposomes remained in circulation, the loaded cGAMP‐Cy5 was rapidly cleared, possibly due to the leakage of cGAMP from liposomes in vivo. By comparison, silicasomes with an amine‐modified MSNP core maintain strong interactions with the negatively charged cGAMP, thereby protecting it during prolonged systemic circulation. To further evaluate the biodistribution of STING agonists delivered via silicasomes, in vivo fluorescence imaging was performed in CT26 tumor‐bearing mice (Figure S13A,B). At 24 h post‐injection, free cGAMP and liposome‐delivered cGAMP exhibited rapid systemic clearance, whereas the cGAMP‐Sili exhibited a 4.5‐fold higher fluorescence signal within the tumor region. Furthermore, in the nanoparticle‐treated group, a strong Cy5 signal persisted in both TDLNs and blood. In contrast, minimal signal was detected in the corresponding tissues of mice treated with free cGAMP and cGAMP‐Lipo (Figure 3G,H, Figure S13C). The cGAMP‐Sili administration markedly enhanced the uptake of STING agonists by intratumoral CD45^+^ immune cells, particularly CD11c^+^MHCII^+^ DCs and CD11b^+^F4/80^+^ macrophages. Previous studies have shown that excessive internalization of STING agonists by T cells can induce apoptosis and impair their immunological function [21, 42]. Accordingly, we analyzed the cellular uptake of ADU‐Sili in T cells and found that minimal uptake occurred in CD3^+^ T cells (Figure 3I, Figures S14 and S15). Immunofluorescence analysis of the tumor sections further confirmed the apparent co‐localization of the Cy5 signal with DCs and macrophages, but not with T cells, supporting the selective cellular internalization and spatial distribution of the nanoparticles within the TME (Figure 3J). These results demonstrate that the silicasome‐based delivery platform enables the efficient and selective delivery of STING agonists to key immune cell subsets in tumors.

### 2.4 Systemic ADU‐Sili Therapy Exerts Potent Antitumor Effects

**FIGURE 4:**
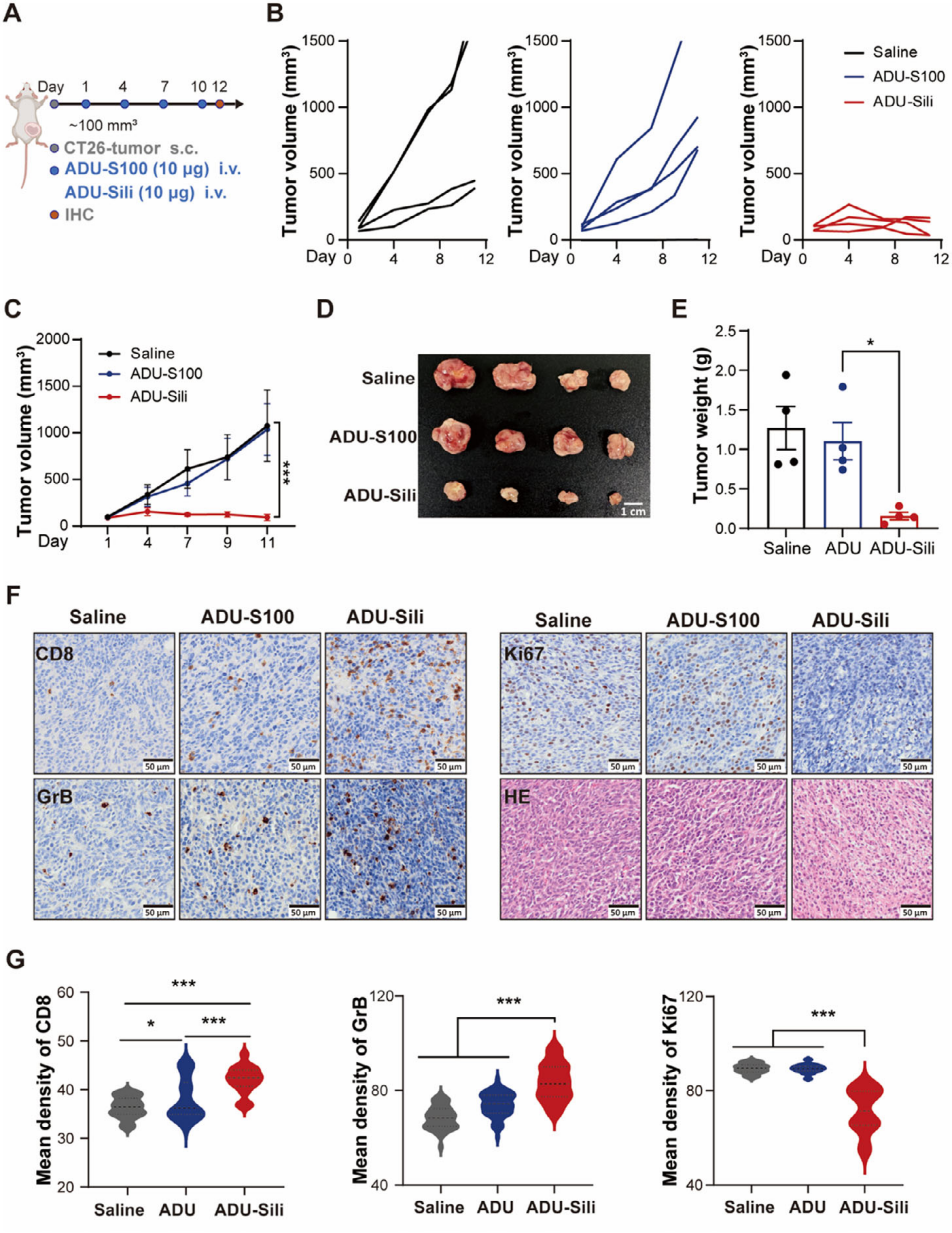
Systemic ADU‐Sili therapy exerts potent anti‐tumor effects. (A) Schematic timeline showing systemic administration of ADU‐S100 or ADU‐Sili (containing 10 µg ADU‐S100 per injection) on days 1, 4, 7, and 10 in CT26 tumor‐bearing mice. (B,C) Individual tumor growth curves (B) and averaged tumor growth curves (C) (n = 4). (D,E) Representative tumor photographs collected 2 days post the final injection (D), and averaged tumor weights (E) (n = 4). Scale bar = 1 cm. (F) Representative IHC staining for CD8, GrB, and Ki67, and H&E staining of tumor sections. Scale bar = 50 µm. (G) Quantitative analysis of the mean density of CD8, GrB, and Ki67. Data represent mean ± SEM. Statistical analysis was performed by one‐way ANOVA with Tukey's multiple comparisons test (E, G), or two‐way ANOVA with Tukey's multiple comparisons test (C). *p < 0.05, **p < 0.01, ***p < 0.001.

While intratumoral injection is commonly adopted due to safety concerns and the limited stability of STING agonists, this route is generally ineffective against inaccessible tumors. To evaluate the anti‐tumor efficacy of STING activation nanoparticles by systemic administration, subcutaneous CT26 tumor‐bearing mice were intravenously (i.v.) treated with saline, free ADU‐S100, or ADU‐Sili (each containing 10 µg of ADU‐S100) (Figure 4A). Compared to the free ADU‐S100 group, administration of ADU‐Sili significantly decreased tumor growth (Figure 4B–E). We further compared the antitumor efficacy of ADU‐S100‐loaded liposomes (ADU‐Lipo) and ADU‐Sili. While ADU‐Lipo demonstrated modest antitumor activity relative to the saline control, ADU‐Sili exhibited significantly superior efficacy (Figure S16). These findings are consistent with the previously observed in vivo PK and biodistribution profiles. It was further demonstrated that even at a low dose (5 µg of ADU‐S100 per administration), the ADU‐Sili exhibited potent antitumor efficacy by suppressing tumor growth and significantly extending survival (Figure S17). Consistently, the expression of Ki67, a marker of cell proliferation, was markedly reduced in tumors from the ADU‐Sili‐treated group compared to the other groups. Furthermore, ADU‐Sili treatment significantly enhanced the expression of CD8 and Granzyme B (GrB) within the TME, whereas the free ADU‐S100 elicited minimal infiltration of effector T cells (Figure 4F,G). In addition, ADU‐Sili administration induced a significant increase in serum cytokine levels, including CXCL10, IL‐6, and TNF‐α, suggesting a systemic immune response. Free ADU‐S100 administration did not elevate cytokine levels, which explains the lack of antitumor efficacy following systemic injection of ADU‐S100 (Figure S18) [43]. These findings suggest robust tumor inhibition and improved antitumor immune response following ADU‐Sili administration.

Meanwhile, we carefully monitored and analyzed the safety of ADU‐Sili during the treatment. Systemic administration of ADU‐Sili only induced a transient body weight loss of approximately 5%, from which the animals rapidly recovered (Figure S19). Analyses of serum chemistry (GLU, AST, ALT, ALP, BUN, CREA, and TBIL) and neurotoxicity markers (AchE) without notable abnormalities indicated that both ADU‐S100 and ADU‐Sili treatments were generally well tolerated (Figure S20). Furthermore, histopathological examination of major organs revealed no abnormal morphological changes, collectively demonstrating the favorable safety profile of these therapies (Figure S21).

### 2.5 ADU‐Sili Augments Anti‐Tumor Immune Response and ICB Therapy

**FIGURE 5:**
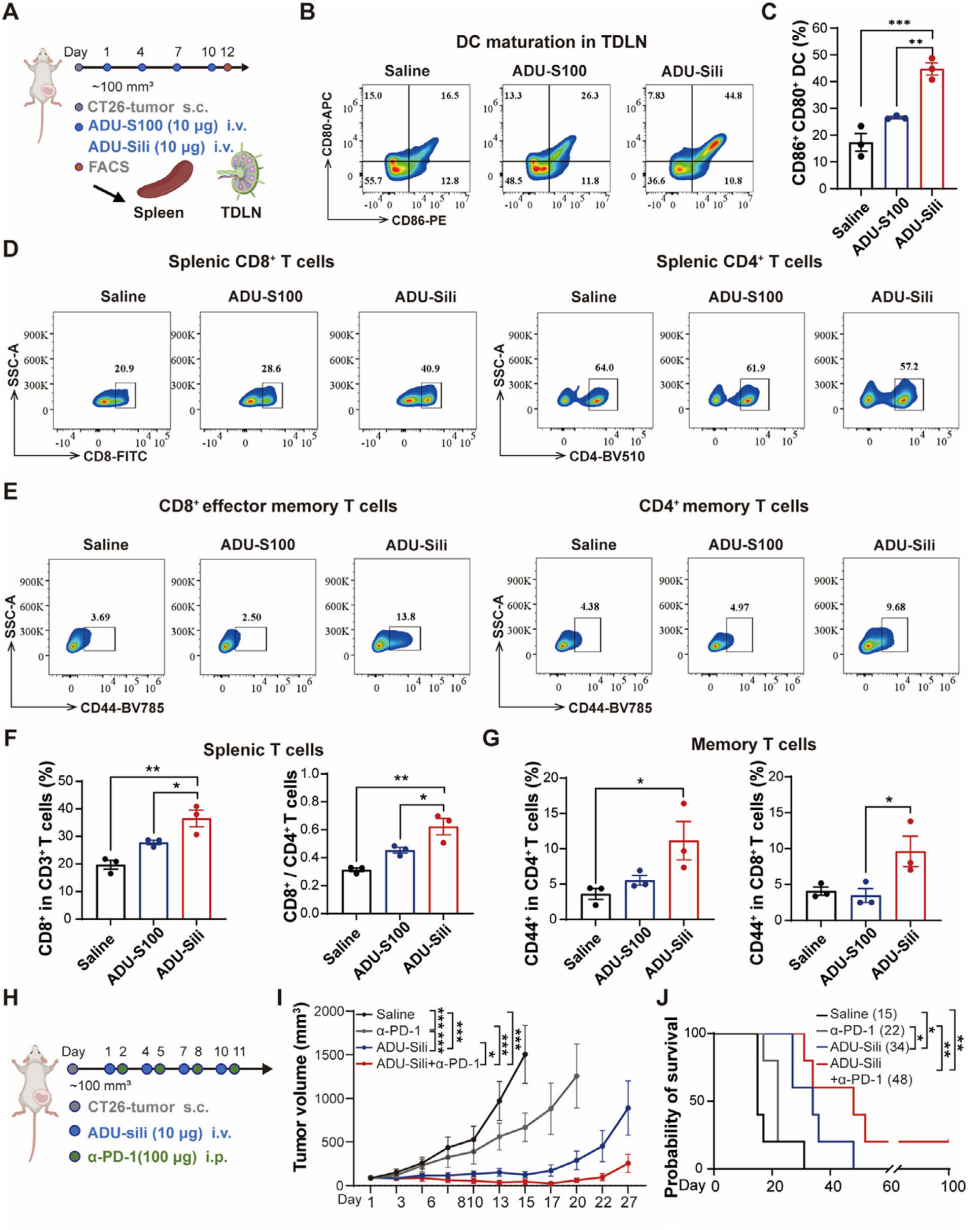
ADU‐Sili augments the anti‐tumor immune response and potentiates immune checkpoint therapy. (A) Schematic timeline of treatment and tissue collection for flow cytometric analysis of TDLNs and spleens in CT26 tumor‐bearing mice. (B,C) Flow cytometry analysis of mature DCs (CD86^+^CD80^+^) in TDLNs (n = 3). (D–G) Splenic T cell response following systemic treatment. Flow cytometry analysis (D) and quantification (F) of CD8^+^ cytotoxic T cells and CD4^+^ T cells in the spleen (n = 3). Representative flow cytometry plots (E) and quantification (G) of CD8^+^ effector memory T cells and CD4^+^ memory T cells (n = 3). (H) Schematic timeline of combined treatment with ADU‐Sili and anti‐PD‐1 antibody in CT26 tumor‐bearing mice. (I) Tumor growth curves of different treatments (n = 5). (J) Survival curves of tumor‐bearing mice receiving the indicated treatments (n = 5). Values in parentheses indicate median survival days. Data represent mean ± SEM. Statistical analysis was performed by one‐way ANOVA with Tukey's (C, G) or Dunnett's (F) multiple comparisons test, or two‐way ANOVA with Dunnett's multiple comparisons test (I), or log‐rank (Mantel‐Cox) test (J). *p < 0.05, **p < 0.01, ***p < 0.001.

To confirm the immunostimulatory effect of ADU‐Sili, we profiled immune cell populations in TDLNs and spleen by flow cytometry (Figure 5A). In the TDLNs, the proportion of mature DCs (CD86^+^CD80^+^ within CD11c^+^ cells) reached 44.7% in the ADU‐Sili group, compared with 17.3% and 26.6% in the saline and free ADU‐S100 group, respectively (Figure 5B,C, Figure S22). Given that DCs' maturation can initiate downstream immune responses by modulating T cell proliferation, we next assessed the frequencies of the splenic CD8^+^ and CD4^+^ T cells. Flow cytometry analysis revealed that the proportion of CD8^+^ cytotoxic T cells was increased by 12.4% in the ADU‐Sili group compared with the free ADU‐S100 group. In contrast, the proportion of CD4^+^ helper T cells remained essentially unchanged (Figure 5D,F, Figure S23). Notably, ADU‐Sili treatment markedly enhanced the population of CD44^+^ effector memory CD8^+^ and CD4^+^ T cells in the spleen, suggesting enhanced memory T cell responses (Figure 5E,G, Figure S23). Collectively, these findings indicate that ADU‐Sili elicits robust immune activation and long‐term immune response, thereby contributing to potent antitumor protection.

Previous studies have demonstrated that STING activation upregulates PD‐L1 expression in tumors [44, 45]. To assess whether ADU‐Sili enhances the efficacy of ICB, we employed the CT26 subcutaneous tumor model, which is known to be resistant to α‐PD‐1 therapy [46, 47]. Mice received intraperitoneal α‐PD‐1 1 day after each ADU‐Sili injection (Figure 5H). While α‐PD‐1 monotherapy exhibited limited antitumor activity, ADU‐Sili alone effectively suppressed tumor growth, and its combination with α‐PD‐1 further enhanced tumor inhibition and prolonged survival (Figure 5I,J, Figure S24). These findings indicate that ADU‐Sili not only exerts antitumor effects as a monotherapy but also augments the efficacy of ICB in a murine colorectal tumor model, demonstrating a synergistic and bidirectional therapeutic benefit.

### 2.6 Systemic ADU‐Sili Administration Suppresses Tumor Progression at Multiple Sites

**FIGURE 6:**
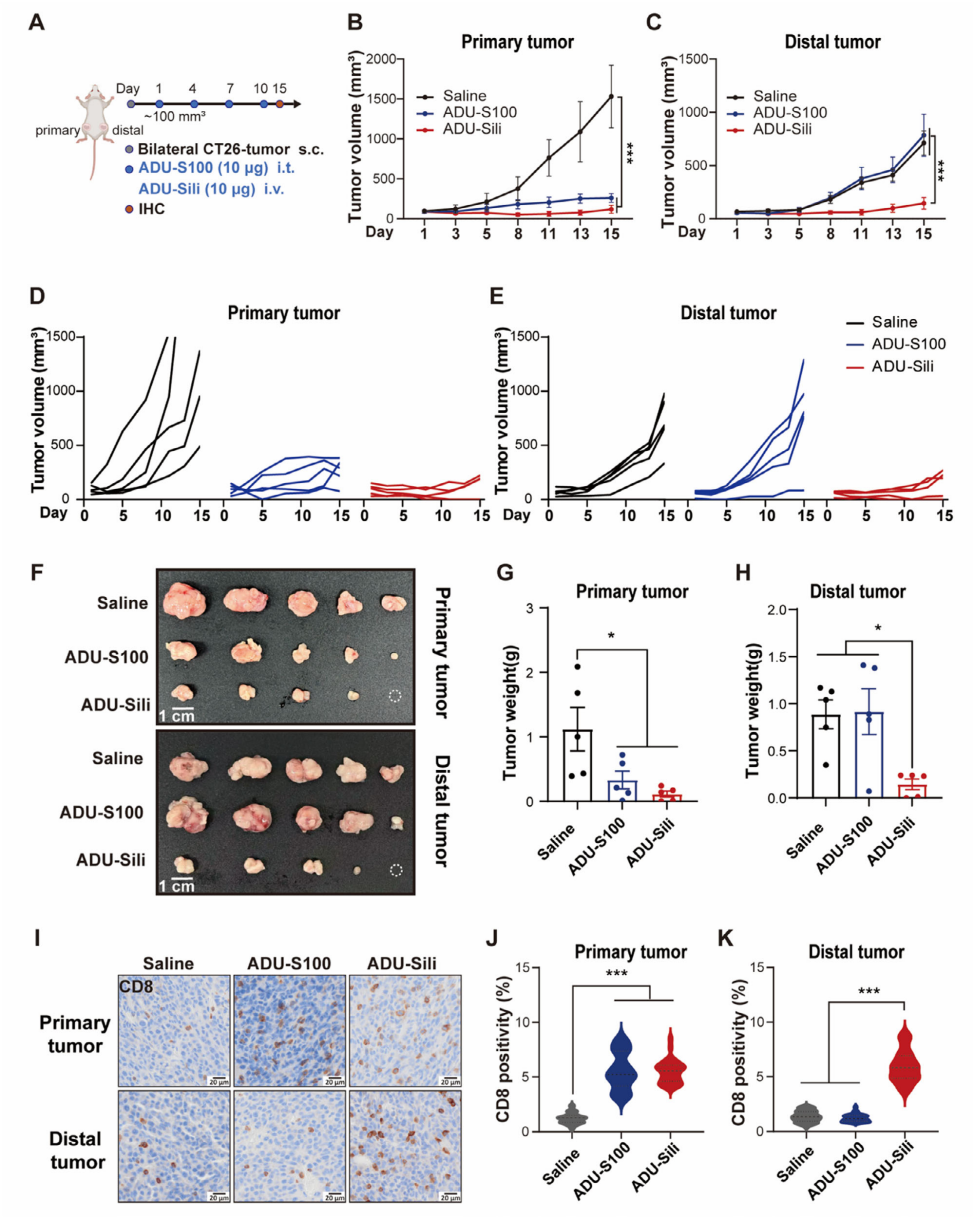
Systemic ADU‐Sili administration suppresses multifocal tumor progression. (A) Schematic timeline of indicated treatment on days 1, 4, 7, and 10 in bilateral CT26 tumor‐bearing mice. (B,C) Tumor growth curves of primary (injected) tumors and distant tumors (n = 5). (D,E) Individual tumor growth curves of primary and distant tumors (n = 5). (F–H) Representative tumor photographs collected 2 days post the final injection and final tumor weights of the primary and distant (n = 5). Scale bar = 1 cm. (I) IHC staining of CD8 in primary and distant tumor sections. Scale bar = 20 µm. (J,K) Quantitative analysis of the CD8 positivity in primary and distant tumors. Data represent mean ± SEM. Statistical analysis was performed by one‐way ANOVA with Tukey's (J, K) or Dunnett's (G,H) multiple comparisons test, or two‐way ANOVA with Tukey's multiple comparisons test (B,C). *p < 0.05, **p < 0.01, ***p < 0.001.

For intratumoral immunotherapy, inducing responses in distal tissues is key to achieving clinically relevant effects. To evaluate the local and systemic antitumor effects of free ADU‐S100 via i.t. administration compared to the i.v. injected ADU‐Sili, a bilateral CT26 tumor model was established, in which mice were subcutaneously inoculated with tumors on both flanks, designating one received i.t. injection as the primary tumor and the other as the distal lesion. Mice received either intratumoral free ADU‐S100, systemic intravenous ADU‐Sili, or saline as a control on days 1, 4, 7, and 10 (Figure 6A). Both ADU‐S100 and ADU‐Sili significantly inhibited the growth of primary tumors compared to saline treatment (Figure 6B,D,F,G). However, in the distal tumors, only systemic ADU‐Sili treatment resulted in a marked reduction in tumor volume. In contrast, intratumoral ADU‐S100 showed limited efficacy at the untreated site (Figure 6C,E,F,H). Immunohistochemical (IHC) analysis further revealed that ADU‐Sili increased CD8^+^ T cell infiltration in both tumors, whereas ADU‐S100 enhanced CD8^+^ T cell infiltration only in the injected tumor (Figure 6I–K). The finding suggests that systemic administration of ADU‐Sili can elicit immune responses and achieve effective tumor suppression in distal tumors, whereas local administration of free ADU‐S100 cannot.

### 2.7 ADU‐Sili Exhibits Antitumor Activity in Orthotopic Tumor Models

**FIGURE 7:**
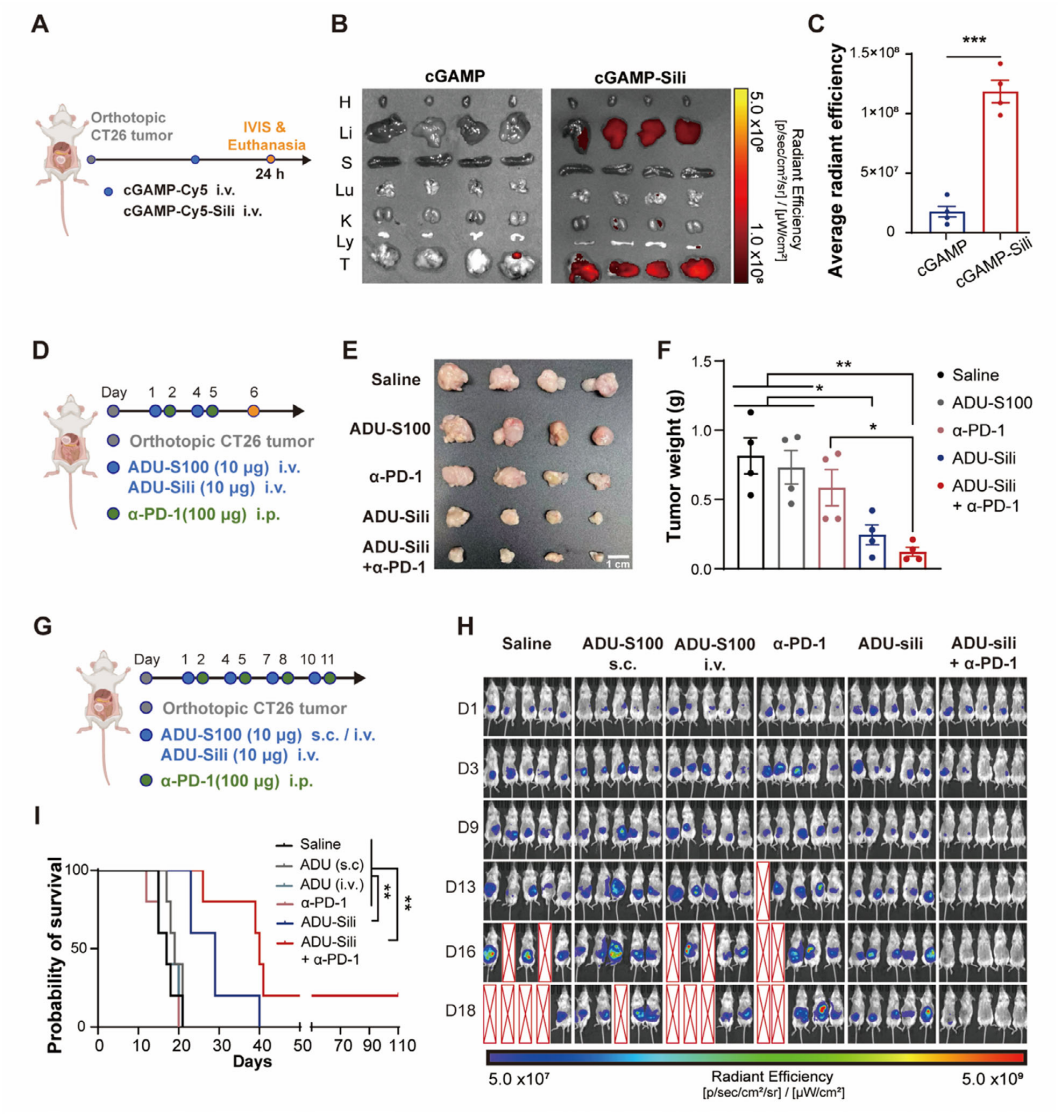
Systemic ADU‐Sili administration exhibits potent antitumor efficacy in an orthotopic tumor model. (A) Schematic illustration depicting the biodistribution of free cGAMP and cGAMP‐Sili in CT26 orthotopic tumor‐bearing mice. (B) Ex vivo images showing the biodistribution of cGAMP‐Cy5 in major organs, including heart (H), liver (Li), spleen (S), lung (Lu), kidney (K), mesenteric lymph node (Ly), and tumor (T). (C) Quantitative analysis of the average radiant efficiency across major organs (n = 4). (D) Experimental timeline illustrating the combination treatment of ADU‐Sili and anti‐PD‐1 antibody in CT26 orthotopic tumor‐bearing mice. Euthanasia was performed after two injections to assess tumor size. (E) Representative tumor photographs of different treatments. Scale bar = 1 cm. (F) Quantitative analysis of tumor weights following treatment (n = 4). (G) Schematic timeline of the survival study involving four administrations of combined ADU‐Sili and anti‐PD‐1 antibody treatment in CT26 orthotopic tumor‐bearing mice. (H) Representative in vivo IVIS bioluminescence images of orthotopic tumor‐bearing mice subjected to various treatments (n = 5). I) Survival curves of orthotopic tumor‐bearing mice under various treatments (n = 5). Data represent mean ± SEM. Statistical analysis was performed by two‐tailed Student's *t*‐test (C), one‐way ANOVA with Tukey's multiple comparisons test (F), or log‐rank (Mantel‐Cox) test (I). *p < 0.05, **p < 0.01, ***p < 0.001.

Given that an orthotopic tumor offers greater clinical relevance by more accurately replicating the native tumor microenvironment, including local vasculature and immune contexture [48, 49, 50]. We established a CT26‐Luciferase orthotopic tumor model to further explore the biodistribution and antitumor efficacy of ADU‐Sili. To assess nanoparticle accumulation, mice bearing orthotopic tumors were intravenously injected with either free cGAMP‐Cy5 or cGAMP‐Sili (DiR‐labelled) 2 weeks post‐implantation (Figure 7A). Ex vivo fluorescence imaging was conducted on major organs and tumors collected 24 h after administration. Results showed that cGAMP‐Sili exhibited approximately a sixfold higher Cy5 fluorescence intensity in tumors compared to the free cGAMP group, which showed negligible signal (Figure 7B,C, Figure S25A). DiR fluorescence was significantly localized to the tumor sites, reflecting preferential tumor accumulation and effective biodistribution of the nanoparticles (Figure S25B,C). These findings highlight the nanoparticle formulation's enhanced tumor accumulation efficiency and support its potential for effective drug delivery in physiologically relevant tumor models. Flow cytometry was further employed to assess the cellular uptake of STING agonist in different cell populations. Notably, cGAMP‐Sili was predominantly internalized by DCs and macrophages within the tumor microenvironment, a pattern that is favorable for the effective activation of STING signaling (Figures S25D,E and S26). The results were consistent with our previous distribution analyses in subcutaneous tumor models.

To assess the therapeutic efficacy of ADU‐Sili in rapidly progressing orthotopic tumors, CT26 tumor tissues were harvested after two intravenous administrations (Figure 7D). ADU‐Sili markedly suppressed orthotopic tumor growth, consistent with findings in the subcutaneous tumor model. In contrast, other groups continued to grow uncontrollably. Combining ADU‐Sili with α‐PD‐1 resulted in the most substantial reduction in tumor volume (Figure 7E,F). To further assess the therapeutic efficacy of STING‐activated silicasome and local injection of free ADU‐S100 in a CT26 orthotopic tumor model, mice were assigned to six groups: saline, ADU‐S100 (subcutaneous injection, s.c.), ADU‐S100 (i.v.), α‐PD‐1, ADU‐Sili (i.v.), and a combination of ADU‐Sili and α‐PD‐1 (Figure 7G). Tumor progression was monitored by bioluminescence (BL) imaging following treatment. The groups receiving saline, s.c. injected ADU‐S100, i.t. injected ADU‐S100 or α‐PD‐1 exhibited a rapid increase in BL intensity, indicating limited antitumor activity. In contrast, ADU‐Sili monotherapy significantly delayed tumor growth relative to other monotherapies. Notably, the combination of ADU‐Sili and α‐PD‐1 achieved the most pronounced suppression of tumor progression and markedly prolonged survival in tumor‐bearing mice (Figure 7I–H). These results highlight the enhanced therapeutic efficacy of combining STING activation with immune checkpoint blockade in orthotopic colorectal cancer. This combinatorial strategy may offer a promising approach for improving clinical outcomes in advanced‐stage tumors.

### 2.8 ADU‐Sili Potentiates Anti‐PD‐1‐Mediated Antitumor Immunity

**FIGURE 8:**
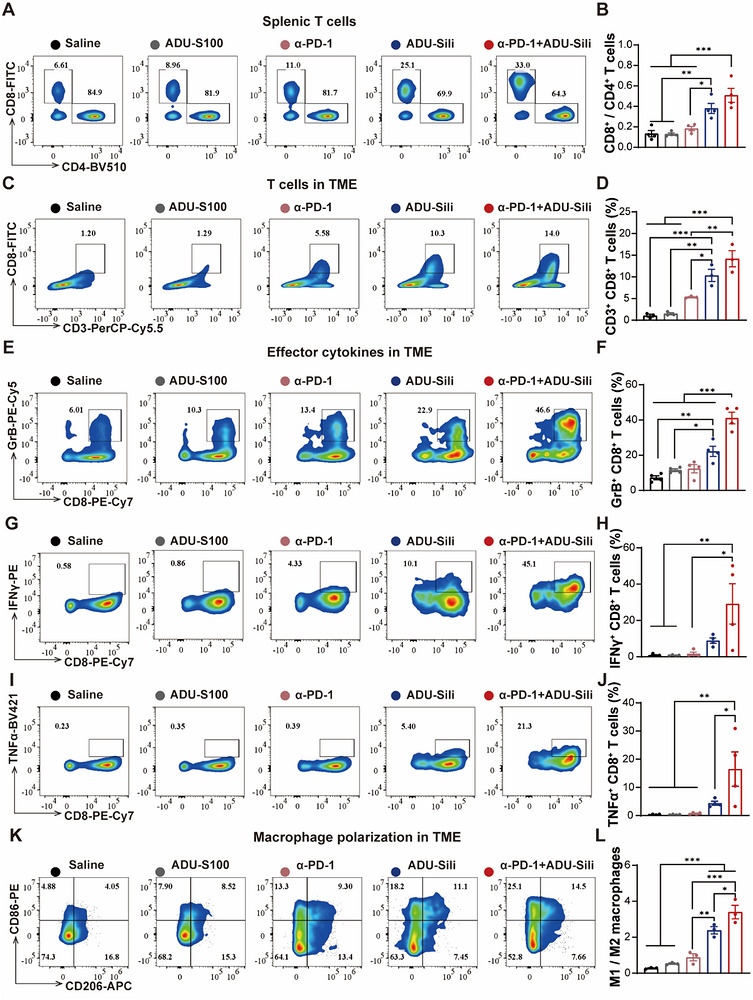
Systemic ADU‐Sili administration potentiates anti‐PD‐1‐mediated antitumor immunity. Flow cytometry analyses were performed after four administrations of the indicated treatments in CT26 orthotopic tumor‐bearing mice. (A) Representative flow cytometry plots of CD8^+^ cytotoxic T cells and CD4^+^ T cells in the spleen. (B) The ratio of CD8^+^ to CD4^+^ T cells (n = 4). (C,D) Representative flow cytometry plots and quantitative analysis of the tumor‐infiltrating CD3^+^CD8^+^ cytotoxic T cells (n = 3). (E,F) Representative flow cytometry plots and quantification of the tumor‐infiltrating GrB^+^ CD8^+^ T cells (n = 4). (G,H) Representative flow cytometry plots and quantification of the tumor‐infiltrating IFNγ^+^ CD8^+^ T cells (n = 4). (I,J) Representative flow cytometry plots and quantification of the tumor‐infiltrating TNFα^+^ CD8^+^ T cells (n = 4). (K) Representative flow cytometry plots of the M1‐like (CD86^+^ CD206^−^) and M2‐like (CD206^+^ CD86^−^) macrophages in TME. (L) The ratio of M1‐like to M2‐like macrophages (n = 3). Data represent mean ± SEM. Statistical analysis was performed by one‐way ANOVA with Tukey's multiple comparisons test. *p < 0.05, **p < 0.01, ***p < 0.001.

To further elucidate the synergistic antitumor immune mechanisms of the nanoparticle formulation, we quantified T‐cell responses in both spleen and tumor tissues. ADU‐Sili monotherapy (25.8%) significantly increased the percentage of splenic CD8^+^ T cells compared with saline (10.1%), ADU‐S100 (10.1%), and α‐PD‐1 monotherapy (13.7%), while the ADU‐Sili plus α‐PD‐1 combination (34.1%) induced the most pronounced expansion of CD8^+^ T cells (Figure 8A,B, Figure S27). Similarly, intratumoral CD8^+^ T cell frequencies were significantly enhanced following ADU‐Sili treatment (10.4%), nearly twofold higher than α‐PD‐1 monotherapy (5.3%), and further increased to 14.2% in the combination group (Figure 8C,D, Figure S28).

As CD8^+^ T cells are the predominant source of effector molecules such as GrB, IFN‐γ, and TNF‐α [51], we next examined functional subsets of tumor‐infiltrating CD8^+^ T cells. The frequency of GrB^+^ CD8^+^ T cells in the ADU‐Sili group was 2.95, 1.87, and 1.72‐fold higher than in saline, ADU‐S100, and α‐PD‐1 groups, respectively, and was further augmented in the combination group (Figure 8E,F, Figure S29). Moreover, ADU‐Sil monotherapy markedly increased the proportion of IFN‐γ^+^ CD8^+^ T cells to 9.1%, compared with saline (0.5%), free ADU‐S100 (0.7%), and α‐PD‐1 (1.6%) groups. And combination treatment with ADU‐Sili and α‐PD‐1 further elevated this percentage to 39.4% (Figure 8G,H, Figure S29). Similarly, ADU‐Sili monotherapy increased the proportion of TNF‐α^+^ CD8^+^ T cells, while the combination group further amplified this effect (Figure 8I,J, Figure S29). These data demonstrate that ADU‐Sili, particularly in combination with PD‐1 blockade, robustly stimulates tumor‐infiltrating CD8^+^ T cells to produce cytotoxic cytokines, thereby enhancing tumor cell killing and suppressing tumor progression.

In addition to T cell activation, ADU‐Sili treatment also reshaped the TAM landscape. The proportions of M1‐like macrophages (F4/80^+^CD86^+^) increased to 19.6% and 23.7% in the ADU‐Sili and combination groups, respectively, which is markedly higher than in the other groups. Conversely, the infiltration of M2‐like macrophages (F4/80^+^CD206^+^) was significantly reduced in both groups (Figure 8K,L, Figure S30). Together, these findings suggest that ADU‐Sili, either as monotherapy or in combination with α‐PD‐1, synergistically enhances antitumor immunity by activating cytotoxic T lymphocytes and reversing macrophage‐mediated immunosuppression.

## 3 Discussion

The cGAS‐STING signaling pathway has emerged as a central axis in tumor immunology due to its ability to sense cytosolic DNA and initiate IFN‐I signaling. Pharmacological activation of STING with CDNs has shown strong preclinical efficacy. Despite compelling preclinical evidence, the clinical trials of STING agonists have proven disappointing to date. The Phase I clinical trial of ADU‐S100 as a monotherapy confirmed its safety and immunostimulatory activity; however, minimal antitumor responses were observed in patients with advanced or metastatic cancers, including those treated with spartalizumab [52, 53]. Similarly, MK‐1454, administered intratumorally, elicited no complete or partial responses. When combined with the PD‐1 monoclonal antibody pembrolizumab, partial responses were observed in only 24% of patients with advanced solid tumors or lymphomas who received treatment [54]. These compounds have primarily been administered intratumorally to achieve high local concentrations while limiting systemic exposure. However, efficacy is modest, predominantly observed in injected tumors, with little impact on inaccessible or metastatic sites.

In this study, we developed a nanoparticle formulation that encapsulates the STING agonist ADU‐S100 for systemic administration, enabling enhanced tumor distribution and potent antitumor efficacy. Across various colorectal cancer models, the ADU‐Sili displayed superior therapeutic performance compared to free ADU‐S100. Sivick et al., reported that intratumoral administration of high doses (100–500 µg) of free ADU‐S100 induces systemic immunity and triggers abscopal tumor regression; however, these doses also cause lymph node damage. Moreover, low doses (10–50 µg) elicit only local inflammation [21]. In contrast, our study demonstrates that systemic delivery of ADU‐Sili effectively suppresses multiple lesions even at low doses, highlighting the critical role of the delivery system in overcoming the dose‐dependent limitation of STING activation. Moreover, ADU‐Sili maintained potent antitumor activity in an orthotopic tumor model that is more clinically relevant. And in the more treatment‐resistant KPC pancreatic tumor model, ADU‐S100 showed only limited antitumor efficacy. In contrast, ADU‐Sili markedly suppressed tumor progression and significantly prolonged survival (Figure S31). These findings underscore the need for systemic delivery and highlight the advantage of silicasome nanocarrier‐mediated STING agonist delivery in overcoming the spatial limitations of current therapeutic approaches.

Mechanistically, ADU‐Sili administration reshaped systemic and tumor immune landscapes. A significant expansion of mature DCs was observed in TDLNs, consistent with the essential role of STING activation in promoting antigen presentation and T cell priming [17]. In the spleen, both CD8^+^ effector T cells and memory T cell populations increased substantially, reflecting the capacity of systemic STING activation to establish durable antitumor immunity. Within tumors, we documented enhanced CD8^+^ T cell infiltration and elevated secretion of cytotoxic cytokines, including GrB, IFN‐γ, and TNF‐α, indicating robust effector functionality. These results are consistent with prior reports demonstrating that STING agonists can transform poorly infiltrated tumors into inflamed microenvironments permissive to T cell‐mediated cytotoxicity. Another key aspect was the reprogramming of TAMs. ADU‐Sili treatment increased the ratio of pro‐inflammatory M1 macrophages while reducing immunosuppressive M2 populations, thus shifting the TME toward an antitumor state. TAM polarization is increasingly recognized as a critical determinant of immunotherapy outcomes, with M1 macrophages amplifying T cell function and M2 macrophages promoting immune evasion [55, 56].

Checkpoint blockade has revolutionized cancer therapy, yet many patients remain non‐responsive due to insufficient T‐cell infiltration and an immunosuppressive tumor microenvironment [2, 4]. Preclinical and clinical studies have shown that STING agonists can enhance DC maturation and promote cross‐priming of CD8^+^ T cells. However, monotherapy with STING agonists often induces only transient tumor regression without durable survival benefits, potentially due to the adaptive upregulation of checkpoint molecules [57, 58]. Therefore, combining STING activation with ICB can provide synergistic efficacy by enhancing T cell recruitment and preventing exhaustion [59, 60]. Consistently, the combination of ADU‐Sili with PD‐1 blockade produced markedly superior tumor control and survival compared with either treatment alone. Our findings indicate that systemic STING activation can overcome PD‐1 resistance by enhancing antigen cross‐presentation, promoting effector T cells infiltration, and remodeling the TME, thereby sensitizing otherwise resistant tumors to ICB. This synergistic effect aligns with prior studies that demonstrated STING pathway activation enhances responses to checkpoint blockade and other immunotherapies.

The superiority of our nanoparticle platform lies in its ability to overcome the limitations of free ADU‐S100. Encapsulation improves stability against nuclease degradation, prolongs circulation, and enhances tumor accumulation [32]. The preferential tumor accumulation of silicasomes likely results from prolonged systemic circulation mediated by PEGylated lipid modification, together with their nanoscale size, which facilitates endothelial transcytosis across tumor vasculature [61, 62]. Moreover, previous studies indicate that MSNPs are biocompatible and safe, gradually hydrolyzing into orthosilicic acid under physiological conditions, which is readily excreted via the kidneys [63]. The silicasome system does not trigger systemic cytokine toxicity but induces only transient increases in IL‐6 and TNF‐α, which rapidly return to normal levels [45]. Compared with conventional liposomes or lipid nanoparticles, the silicasome platform combines a MSNP core with a lipid bilayer coating, enabling efficient loading of highly hydrophilic and negatively charged STING agonists while improving colloidal stability and circulation to enhance tumor accumulation [33]. These properties extend the applicability of STING agonists beyond intratumoral delivery, enabling treatment of metastatic and inaccessible tumors. Nevertheless, further studies are warranted to comprehensively evaluate long‐term in vivo safety and biodegradation, which will be critical for future clinical translation.

While our data indicate a favorable therapeutic index, optimizing the nanoparticle composition, size, and surface modification may further enhance tumor targeting and reduce off‐target immune activation [64]. Additionally, intertumoral heterogeneity in STING pathway activity may influence responsiveness, underscoring the need to identify predictive biomarkers such as baseline STING expression. Taken together, our study demonstrates that systemic administration of ADU‐Sili can overcome the pharmacological and biological barriers of free ADU‐S100, remodel the immune microenvironment, and synergize with ICBs. By enabling tumor‐wide immune activation, this approach represents a significant step toward the clinical translation of STING agonist‐based therapies.

## 4 Conclusion

Our study demonstrates that silicasome‐mediated systemic delivery of STING agonists offers a promising approach to overcome the limitations of free CDNs. Compared with conventional liposomes, silicasomes achieved higher STING agonist loading efficiency and enabled prolonged systemic circulation of STING agonists through the engineered MSNP core. The silicasome‐based delivery system improved tumor accumulation, promoted dendritic cell maturation, expanded effector and memory CD8^+^ T cells, enhanced cytotoxic cytokine release, and shifted TAM polarization toward an antitumor state. Systemic administration further enabled the suppression of orthotopic and distal tumors, which was not achievable by intratumoral injection. When combined with PD‐1 blockade, the treatment produced synergistic effects, underscoring the translational potential of ADU‐Sili in combinatorial immunotherapy. Overall, these findings highlight the importance of systemic delivery and rational nanocarrier design for the clinical development of STING agonists.

## 5 Materials and Methods

### 5.1 Materials

RPMI‐1640 medium (catalog no. c11875500bt) and fetal bovine serum (FBS, catalog no. 10099141) were purchased from Gibco. Granulocyte‐macrophage colony‐stimulating factor (GM‐CSF) was obtained from GenScript (catalog no. Z03300‐10). Primers were purchased from Tsingke Biotech, and the OVA_257–264_ peptide was purchased from GLPBIO (catalog no. GC33448); their sequences are shown in the information (Tables S1–S2). The collagenase type IV was purchased from Gibco (catalog no. 17104019). The cGAMP‐Cy5 was obtained from Biolog (catalog no. C271‐001). ELISA kits for IFN‐β were purchased from BioLegend (catalog no. 439404), and for CXCL10, TNF‐α, and IL‐6 were purchased from Abclonal (catalog no. RK00056, RK04595, RK00008). Antibodies used for immunoblotting were purchased from Cell Signaling Technology, including anti‐pTBK1 antibody (catalog no. 5483), anti‐pIRF3 antibody (catalog no. 29047), anti‐pSTING antibody (catalog no. 50907), anti‐TBK1 antibody (catalog no. 38066), anti‐STING antibody (catalog no. 13647), and HRP‐conjugated anti‐rabbit secondary antibody (catalog no. 7074). Anti‐GAPDH antibody (catalog no. A19056) was purchased from Abclonal. Antibodies used for IHC and immunofluorescence were purchased from Abcam, including anti‐CD8 (catalog no. ab209775), anti‐Granzyme B (catalog no. ab255598), anti‐Ki67 (catalog no. ab16667), and anti‐CD11c (catalog no. ab254183). Anti‐F4/80 (catalog no. 123102) was purchased from BioLegend. The following flow cytometry antibodies were purchased from BioLegend, including APC‐Cy7‐CD11c (catalog no. 117324), BV421‐CD80 (catalog no. 104725), PE‐MHCII (catalog no. 107608), APC‐H‐2K^b^ bound to SIINFEKL (catalog no. 141606), PerCP‐Cy5.5‐CD3 (catalog no. 100218), FITC‐CD8 (catalog no. 100705), PE‐Cy5‐Granzyme B (catalog no. 372226), PE‐IFN‐γ (catalog no. 505807), BV421‐TNF‐α (catalog no. 506327), APC‐CD206 (catalog no. 141708), FITC‐CD80 (catalog no. 104706), BV650‐MHCII (catalog no. 107641), BV605‐CD45 (catalog no. 103139), BV510‐CD4 (catalog no. 100559), PE‐Cy7‐F4/80 (catalog no. 123114), and BV785‐CD44 (catalog no. 103059). The following flow cytometry antibodies were purchased from BD Biosciences, including BV421‐CD11c (catalog no. 565452), APC‐CD80 (catalog no. 560016), BV650‐CD86 (catalog no. 743214), AF700‐CD45 (catalog no. 560510), BV650‐CD11b (catalog no. 563402), BV421‐F4/80 (catalog no. 565411), BV605‐Ly6C (catalog no. 563011), BB515‐CD11b (catalog no. 564454), PE‐Cy7‐CD8 (catalog no. 552877), and CD86‐PE (catalog no. 553692). All the chemicals were purchased for direct use without further purification.

### 5.2 Cell Culture

The murine colon carcinoma cell line CT26‐Luciferase was kindly provided by the National Center for Nanoscience and Technology, China (Cellverse, catalog no. iCell‐0061a). The human monocytic reporter cell line THP1‐Dual (RRID: CVCL_X599) was purchased from InvivoGen (catalog no. thpd‐nfis). CT26‐Luciferase and THP1‐Dual cells were maintained in RPMI‐1640 supplemented 10% FBS and 1% penicillin‐streptomycin. All cells were incubated at 37°C in a humidified atmosphere containing 5% CO_2_. All cell lines were routinely authenticated as required and tested negative for mycoplasma and other microbial contamination prior to use.

### 5.3 Synthesis of MSNPs and ADU‐S100 Loading

Bare MSNPs were synthesized using a sol‐gel approach as previously described [33]. To obtain aminated MSNPs, bare MSNPs were dispersed in ethanol, followed by the addition of 3‐aminopropyltriethoxysilane (APTES) at 10% (APTES volume/MSNP mass, µL/mg) relative to the MSNPs. The mixture was stirred overnight at 80°C. Afterward, any residual silane coupling agent was removed. The resulting MSNP‐NH_2_ were dispersed in ethanol.

To prepare ADU‐MSNPs, 200 µg of ADU‐S100 was added to 10 mg of MSNP‐NH_2_ in diethylpyrocarbonate (DEPC)‐treated water, followed by bath sonication for 5 min. The mixture was then centrifuged at 15 000 rpm for 20 min. The loading capacity of ADU‐S100 was quantified by measuring the residual amount in the supernatant using a differential method; the pellet was then dispersed in DEPC‐treated water. The ADU‐MSNPs were further coated with a lipid membrane by mixing 20 mg of liposomes (containing 26.6 mg DSPC, 8.7 mg cholesterol, and 4.7 mg DSPE‐PEG2000), followed by bath sonication. Unincorporated liposomes were removed, and the resulting product was termed ADU‐Sili.

To fabricate ADU‐Lipo, a lipid mixture (20 mg) consisting of DSPC, cholesterol, and DSPE‐PEG2000 was dissolved in chloroform and subsequently subjected to rotary evaporation to form a thin lipid film. The dried lipid film was hydrated with an ADU‐S100 solution (200 µg/mL) under constant stirring in a sonication bath. The resulting suspension was extruded 17 cycles using a mini‐extruder (Avanti) equipped with a 0.1 µm pore‐sized polycarbonate membrane (Cytiva). To eliminate free ADU‐S100, the samples were purified using a 100 kDa molecular weight cut‐off (MWCO) centrifugal filter (Millipore). The loading capacity of ADU‐S100 was determined by a differential method that quantified the unencapsulated drug. For experiments involving fluorescently labeled CDN‐loaded nanoparticles, cGAMP‐Cy5 was used as a substitute for ADU‐S100.

### 5.4 Characterization of Nanoparticles

The particles' hydrodynamic size and ζ potential were measured using a particle and molecular charge analyzer (Malvern, Zetasizer Nano ZS ZEN3600). Particle size measurements were conducted by diluting the nanoparticles in deionized water, while potentiometric measurements were performed by diluting the nanoparticles in 10 mM NaCl. The final product was visualized using Cryo‐transmission electron microscopy (Cryo‐TEM) to confirm the uniformity and integrity of the coated lipid bilayer.

### 5.5 THP1‐Dual Reporter Assay

THP1‐Dual cells (InvivoGen) were utilized to evaluate the activation of the interferon regulatory factor 3 (IRF3) pathway by measuring luciferase activity. The cells were seeded into 96‐well plates at a density of 50 000 cells per well. ADU‐S100 and ADU‐MSNPs were introduced at concentrations ranging from 0 to 2 µM. After 24 or 48 h of incubation at 37°C, luciferase activity was quantified using the QUANTI‐Luc assay (InvivoGen) on a Spark multimode microplate reader (TECAN).

### 5.6 Activation of the cGAS‐STING Pathway in BMDCs

Mouse bone marrow‐derived dendritic cells (BMDCs) were isolated and cultured in a GM‐CSF‐containing medium. Fresh medium was added on days 3, 5, and 7. After 7 days, BMDCs were harvested for further use. To investigate cGAS‐STING pathway activation, BMDCs were then incubated with ADU‐S100 and ADU‐MSNPs at a concentration of 1 µM for 6 h. Cells were lysed in RIPA buffer supplemented with phosphatase and protease inhibitors. Protein extracts were separated by SDS‐PAGE and transferred to PVDF membranes. After blocking with 5% bovine serum albumin (BSA) for 1 h, membranes were incubated overnight at 4°C with primary antibodies, including anti‐pTBK1, anti‐pIRF3, anti‐pSTING, anti‐TBK1, and anti‐STING (1:1000), and anti‐GAPDH (1:5000). Membranes were then washed and incubated with HRP‐conjugated anti‐rabbit secondary antibodies (1:2000) for 1 h at room temperature. Protein bands were then detected using the Amersham ImageQuant 800 system (Cytiva).

### 5.7 Detection of IFNβ Expression Levels in BMDCs

To evaluate the expression of IFN‐β, BMDCs were subsequently exposed to ADU‐S100 and ADU‐MSNPs at a concentration of 1 µM. After 24 h of treatment, the culture supernatant was collected separately. IFN‐β levels in the supernatant were determined using a mouse IFN‐β ELISA kit.

Following treatment, cells were collected for real‐time quantitative PCR (RT‐qPCR. RNA was extracted using FreeZol (Vazyme), and cDNA synthesis was carried out with the All‐in‐One First‐Strand cDNA Synthesis Kit (TransGen Biotech). Then, cDNA was combined with primers and Taq Pro Universal SYBR qPCR Master Mix (Vazyme) and amplified using a CFX96 Touch Thermal Cycler (Bio‐Rad). Relative expression levels of *Ifnb* were determined by the ∆∆Ct method and normalized to 18S rRNA.

### 5.8 In Vitro Evaluation of BMDCs Maturation

BMDCs were plated in 12‐well plates and cultured overnight. ADU‐S100 or ADU‐MSNPs were then introduced to the cells at a concentration of 1 µM. After 24 h of incubation, the cells were harvested and washed with FACS buffer (2% FBS in PBS), then stained with antibodies including APC‐Cy7‐CD11c, BV421‐CD80, PE‐CD86, and BV650‐MHCII. Subsequently, the stained cells were washed, resuspended in FACS buffer, and analyzed using a CytoFLEX LX flow cytometer (Beckman Coulter). Data were then processed using FlowJo v.10.8.1 software.

### 5.9 In Vitro BMDC Antigen Presentation Assay

BMDCs were seeded in 12‐well plates and cultured overnight. ADU‐S100 or ADU‐MSNPs were then introduced at a concentration of 1 µM. After 24 h of incubation, OVA_257–264_ peptide (1 µg/mL) was added and treated for an additional 2 h. The cells were subsequently collected, washed, and stained with antibodies, including BV421‐CD11c and APC‐H‐2Kb bound to SIINFEKL. Then, stained cells were analyzed using a CytoFLEX LX flow cytometer.

### 5.10 In Vitro Assessment of Cellular Uptake in BMDCs

To quantify and visualize the cellular uptake of STING agonist‐loaded MSNPs, cGAMP‐Cy5 was utilized as a mimic of ADU‐S100. BMDCs were seeded in 12‐well plates and cultured overnight. Then, cells were exposed to free cGAMP‐Cy5 or cGAMP‐MSNPs for 6, 12, and 24 h. Cells were collected, and fluorescence was quantified via flow cytometry. To investigate the subcellular localization of internalized cGAMP‐Cy5, BMDCs were cultured in glass‐bottom dishes and incubated with free cGAMP‐Cy5 or cGAMP‐MSNPs for 12 and 24 h. Nuclei were stained with DAPI, and confocal images were acquired using an LSM900 confocal microscope (Zeiss) and analyzed using ZEN software.

### 5.11 In Vivo STING Agonist Distribution Assay

Free cGAMP‐Cy5, cGAMP‐Sili, and cGAMP‐Lipo were employed to investigate the biodistribution of the STING agonist. The formulations above were administered via tail vein injection to CT26 tumor‐bearing mice. Biodistribution was assessed at the following time points (0, 4, 12, and 24 h or 10 min, 2 h, 6 h, 12 h, 24 h) using an In Vivo Imaging System (IVIS, PerkinElmer), with the fluorescence signal of cGAMP‐Cy5 measured at excitation/emission wavelengths of 640/700 nm or the DiR signals measured at 740/790 nm. At 24 h post‐injection, mice were euthanized, and the fluorescence signals in excised organs were analyzed to determine tissue‐specific accumulation.

To investigate drug distribution within the tumor microenvironment, a portion of the excised tumors was embedded in OCT compound, sectioned into 5 µm slices, and stained with the following antibodies: anti‐F4/80, anti‐CD11c, and anti‐CD8. The stained sections were imaged using a pathology panoramic scanning system (AKOYA Vectra Polaris) for spatial analysis. Remaining tumor tissues were enzymatically digested with DNase I (0.1 mg/mL) and collagenase IV (1 mg/mL) to generate single‐cell suspensions, which were stained and analyzed by flow cytometry to quantify phagocytosed cGAMP‐Cy5 across different cell populations. The antibodies used for staining included AF700‐CD45, BV421‐CD11c, PE‐MHCII, PerCP‐Cy5.5‐CD3, BV421‐F4/80, BV650‐CD11b, and BV605‐Ly6C. Data were captured using a CytoFLEX LX cytometer (Beckman Coulter) and subsequently analyzed with FlowJo software (v10.8.1).

### 5.12 In Vivo Antitumor Studies

Animal experiments were performed in accordance with institutional and national guidelines for laboratory animal care and were approved by the Animal Center of the Hangzhou Institute of Medicine, Chinese Academy of Sciences (AP2024‐08‐0097). The study utilized female BALB/c mice and C57BL/6 mice aged 6–8 weeks, purchased from the Zhejiang Experimental Animal Center. Mice were euthanized by CO_2_ inhalation when the larger tumor exceeded 1500 mm^3^ or when they exhibited severe morbidity, such as significant weight loss or severe ascites.

For the establishment of the CT26 murine subcutaneous tumor model, 5 × 10^5^ CT26 cells were injected subcutaneously into the right back flank. When tumor volumes approached ∼100 mm^3^ (volume = width^2^ × length × 0.5), mice were randomly assigned to experimental groups. The designated drugs or formulations (ADU‐S100, ADU‐Sili) were injected via the tail vein at a dose of 10 µg/200 µL every 3 days; additionally, α‐PD1 (100 µg/dose, 200 µL) was administered intraperitoneally 1 day after the drugs above, with a total of 4–5 treatments. Tumor progression was measured at 2–4‐day intervals.

The CT26 bilateral subcutaneous tumor model was generated by subcutaneously injecting 5 × 10^5^ CT26 cells into both flanks. Once the larger of the two tumors reached approximately 100 mm^3^, ADU‐S100 was administered intratumorally into the larger tumor (the primary tumor), while the untreated tumor was considered the distal tumor. Concurrently, ADU‐Sili was delivered via intravenous injection every 3 days at a dose of 10 µg/200 µL, with a total of 4 treatments. The growth of both tumors was monitored every 2–4 days.

To establish the orthotopic CT26 colorectal tumor model, subcutaneous CT26‐Luc tumors were excised and cut into 1 mm^3^ pieces. The upper end of the cecum was gently scraped to induce minor bleeding, and the tumor pieces were then attached to the bleeding site using biological adhesive. Mice received postoperative care, and tumor progression was monitored using the IVIS imaging system. Mice were anesthetized with isoflurane before in vivo imaging. The designated drugs or formulations (ADU‐S100, ADU‐Sili) were administered via intravenous injection at a dose of 10 µg/200 µL every 3 days. Additionally, α‐PD1 (100 µg/dose, 200 µL) was injected intraperitoneally 1 day after the administration of the drugs above, for a total of 4 treatments. Tumor progression was imaged at 3–4‐day intervals. The KPC subcutaneous tumor model was generated by injecting 2 × 10^6^ KPC pancreatic cancer cells into the flank.

### 5.13 Immunohistochemistry and Hematoxylin‐Eosin Staining

48 h after the last administration, tumors were collected, fixed in 10% formalin, dehydrated in 70% ethanol, embedded in paraffin, and sliced into 5 µm sections. For immunohistochemistry (IHC), the following antibodies were used: anti‐CD8, anti‐Granzyme B, and anti‐Ki67. IHC was performed using the Leica Bond RXm immunohistochemistry and in situ hybridization staining system. Images were acquired using an Olympus VS200 pathology slide scanner and analyzed with ImageJ software. Tissue sections were stained with hematoxylin‐eosin (H&E) using an automatic dyeing system (Sakura, DRS‐Prisma‐P‐JCS&Film‐JC2). Imaging was performed on an Olympus VS200, and the images were processed with ImageJ.

### 5.14 In Vivo Immune Response Analysis

24 h after the last injection, tumors were harvested, sectioned into small pieces, and subjected to enzymatic digestion with DNase I (0.1 mg/mL) and collagenase IV (1 mg/mL) to generate single‐cell suspensions. Following digestion, the cell suspension was passed through a 70 µm strainer and subsequently incubated with anti‐CD16/32 to block nonspecific binding. The cells were subsequently stained with antibodies, including AF700‐CD45, PerCP‐Cy5.5‐CD3, FITC‐CD8, PE‐Cy5‐Granzyme B, PE‐IFN‐γ, BV421‐TNF‐α, PE‐Cy7‐F4/80, APC‐CD206, BB515‐CD11b and CD86‐PE.

TDLNs were collected, dissected, and digested into a single‐cell suspension. The suspension was then filtered through 70 µm strainers, washed, and blocked with anti‐CD16/32. Subsequently, cells were stained with antibodies: AF700‐CD45, BV421‐CD11c, FITC‐CD80, BV650‐MHCII, and PE‐CD86.

Collected spleens were gently dispersed to prepare single‐cell suspensions, filtered using a 70 µm strainer, and treated to lyse red blood cells. Cells were subsequently incubated with anti‐CD16/32 and stained with antibodies including AF700‐CD45, BV605‐CD45, PerCP‐Cy5.5‐CD3, BV510‐CD4, FITC‐CD8, and BV785‐CD44.

### 5.15 Statistical Analysis

Data are shown as mean ± SEM and analyzed with GraphPad Prism 10. Comparisons between two groups were conducted using an unpaired Student's *t*‐test, one‐way ANOVA was applied for multiple group comparisons, and two‐way ANOVA was used for experiments with two independent variables. *p < 0.05, **p < 0.01, ***p < 0.001. Data from mice that died accidentally during the experiment, for reasons unrelated to treatment, were excluded from the final analysis. “n” represents biological replicates.

## Author Contributions

## Conflicts of Interest

The authors declare no conflicts of interest.

## Supporting information




**Supporting File**: advs75108‐sup‐0001‐SuppMat.pdf.

## Data Availability

The data that support the findings of this study are available in the material of this article.;
