# Supplementary material for: Nano‐Enabled Systemic Delivery of STING Agonist by Engineered Silicasome for Potent Antitumor Immunotherapy
Source: Adv Sci (Weinh). 2026 Apr 9;13(34):e23722. doi: 10.1002/advs.202523722 (PMC13285114; doi:10.1002/advs.202523722)
Supplement: Supplementary file 1 — Supporting File: advs75108‐sup‐0001‐SuppMat.pdf. [file ADVS-13-e23722-s001.pdf]

Supporting Information

Nano-enabled Systemic Delivery of STING Agonist by Engineered Silicasome for Potent Antitumor Immunotherapy

Wenjing Zhou[1, 2], Yuting Li[2, *], Xudong Sun[3], Honghong Yang[2], Xiayu Shi[2], Lijia Yao[2], Jinhong Jiang[2], Erli Wang[2], Haoyang Wang[2, 4], Yi Wei[2], Xiangsheng Liu[2, *]

[1]School of Life Sciences, Tianjin University, Tianjin 300072, China;

[2]Zhejiang Cancer Hospital, Zhejiang Key Laboratory of Functional Nucleic Acids for Basic and Clinical Application, Hangzhou Institute of Medicine, Chinese Academy of Sciences, Hangzhou 310018, China;

[3]Fuyang Institute of Zhejiang Chinese Medical University, Hangzhou 311400, China;

[4]College of Pharmaceutical Science, Zhejiang University of Technology, Zhejiang, Hangzhou 310014, China

*To whom correspondence should be addressed:

liuxs@him.cas.cn

liyuting@him.cas.cn

Material and methods

Materials

Anhydrous solvents, including acetonitrile, pyridine, and triethylamine, were purchased from J&K Scientific. Phosphoramidite reagents were purchased from Bide Pharmatech. DMEM/F12 medium (catalog no. c11320033bt) and fetal bovine serum (FBS, catalog no. 10099141) were purchased from Gibco.

Cell culture

The murine pancreatic cancer cell KPC was kindly provided by the College of Pharmaceutical Sciences, Zhejiang University provided by Dr. Tingbo Liang's group. KPC cells used in this study does not have a Research Resource Identifier (RRID) because it is not commercially available. KPC cells were cultured in DMEM/F12 containing 10% FBS and 1% penicillin-streptomycin. KPC cells were incubated at 37 °C in a humidified atmosphere containing 5% CO_2. KPC cells were routinely authenticated as required and tested negative for mycoplasma and other microbial contamination prior to use.

Preparation of ADU-S100

To a solution of compound **1,** DMT-2'O-TBDMS-rA (bz) phosphoramidite (4.5 g, 4.55 mmol) in 23 mL of acetonitrile, H_2O (162 μL, 9 mmol), and pyridine trifluoroacetate salt (1.04 g, 5.4 mmol) were added. The mixture was stirred at room temperature for 1 min, then 22.5 mL of tert-butylamine (t-$BuNH_2$) was added. After 10 min, the solvent was removed in vacuo, and the mixture was azeotroped with acetonitrile three times. Then, the mixture was dissolved in 54 mL of dichloromethane (DCM) containing 0.81 mL of H_2O, followed by the addition of 54 mL 6% dichloroacetic acid (DCA) solution in DCM under an ice bath. The reaction was allowed to warm to room temperature and stirred for 20 min. Upon completion, the reaction was quenched by 7 mL of pyridine and concentrated in vacuo to afford the crude compound **2** without purification.

Compound **2** was azeotroped three times with 30 mL of dry acetonitrile, leaving 15 mL in the last time. The dried solution of 3'-OTBS rA phosphoramidite (compound **3**, 5.6g, 5.9 mmol) in dry acetonitrile was then added using a dry syringe. After 5 min, 3-

[(dimethylaminomethylene) amino]-3H-1,2,4-dithiazole-5-thione (DDTT, 1.2 g, 5.9 mmol) was added, and the reaction was continued for 30 min. After completion, the solvent was removed, and the compound was redissolved in 100 mL of DCM. This solution was then treated with 0.5 mL of H_2O and a 6% DCA solution in DCM (54 mL). After 20 min, the reaction was quenched by 42 mL of pyridine and concentrated in vacuo to give the crude compound **4**. The residue was azeotroped with 90 mL of pyridine three times, leaving 50 mL in the last time, followed by the addition of 2-chloro-5,5-dimethyl-1,3,2-dioxaphosphorinane 2-oxide (DMOCP, 2.02 g, 11 mmol) at ambient temperature. After 30 min, 2 mL H_2O quenched the reaction, and 3H-1,2-benzodithiol-3-one (794 mg, 4.7 mmol) was added immediately. After 20 minutes, the reaction mixture was poured into a 500 mL solution of 3% $NaHCO_3$ and stirred for 10 minutes. The mixture was then extracted with a 1:1 ethyl acetate/methyl tert-butyl ether (EtOAc/MTBE) mixture three times. The organic layers were combined, washed with aqueous NaCl, dried over Na_2SO_4, filtered, and concentrated under reduced pressure. The residue was purified by silica gel column chromatography (DCM: methanol = 80:1 to 50:1) to give the diastereomers **5**.

Compound **5** (500 mg, 0.424 mmol) was dissolved in 20 mL methylamine/methanol ($MeNH_2$/MeOH) solution and stirred at 30 °C for 3 hours. Upon completion, the solvent was removed under reduced pressure, and the product was purified by HPLC using a Gilson Venusil XBP C18 column (10 μm, 100 Å, 21.2 × 250 mm) to afford compound **6**.

To a solution of compound **6** (65 mg, 0.07 mmol) in 0.7 mL anhydrous pyridine and 0.7 mL triethylamine (Et_3N), 0.21 mL triethylamine trihydrofluoride ($Et_3N\cdot 3HF$) was added. After stirring at 50 °C for 3 h, the mixture was poured into a cold 1 M triethylammonium acetate buffer, and the product ADU-S100 was purified by HPLC using a Waters XBridge C18 column. ^{1}H NMR (400 MHz, D_2O) δ = 8.37 (s, 1H), 8.10 (s, 1H), 7.99 (s, 1H), 7.90 (s, 1H), 6.10 (d, *J* = 8.4 Hz, 1H), 5.99 (d, *J* = 1.6 Hz, 1H), 5.31 (td, *J* = 8.6, 4.0 Hz, 1H), 5.00 (td, *J* = 8.2, 4.5 Hz, 1H), 4.81 (d, *J* = 4.0 Hz, 1H), 4.65 (m, 1H), 4.48 - 4.36 (m, 2H), 4.31 - 4.23 (m, 2H), 4.22 - 4.14 (m, 1H), 3.99 (dd, *J* = 11.8, 4.7 Hz, 1H). ^{31}P NMR (162 MHz, D_2O) δ = 54.75, 52.32. MS (ESI): m/z 689 ($[M + H]^-$).

***In vitro* evaluation of BMDCs maturation**

BMDCs were plated in 12-well plates and cultured overnight. MSNP, or MSNP-NH_2 were then introduced to the cells at a concentration of 100 μg/mL. After 24 hours of incubation, the cells were harvested and washed with FACS buffer (2% FBS in PBS), then stained with antibodies including APC-Cy7-CD11c, BV421-CD80, and PE-CD86. Subsequently, the stained cells were washed, resuspended in FACS buffer, and analyzed using a CytoFLEX LX flow cytometer (Beckman Coulter). Data were then processed using FlowJo v.10.8.1 software.

Table S1. Primer sequences

Gene	Sequences F (5'-3')	Sequences R (5'-3')
Ifnb	CGTGGGAGATGTCCTCAACT	AGATCTCTGCTCGGACCACC
18S	GTAACCCGTTGAACCCCATT	CCATCCAATCGGTAGTAGCG

Table S2. Peptide sequences

Gene	Sequence
$OVA_{257\text{-}264}$	SIINFEKL

Supplementary Figures

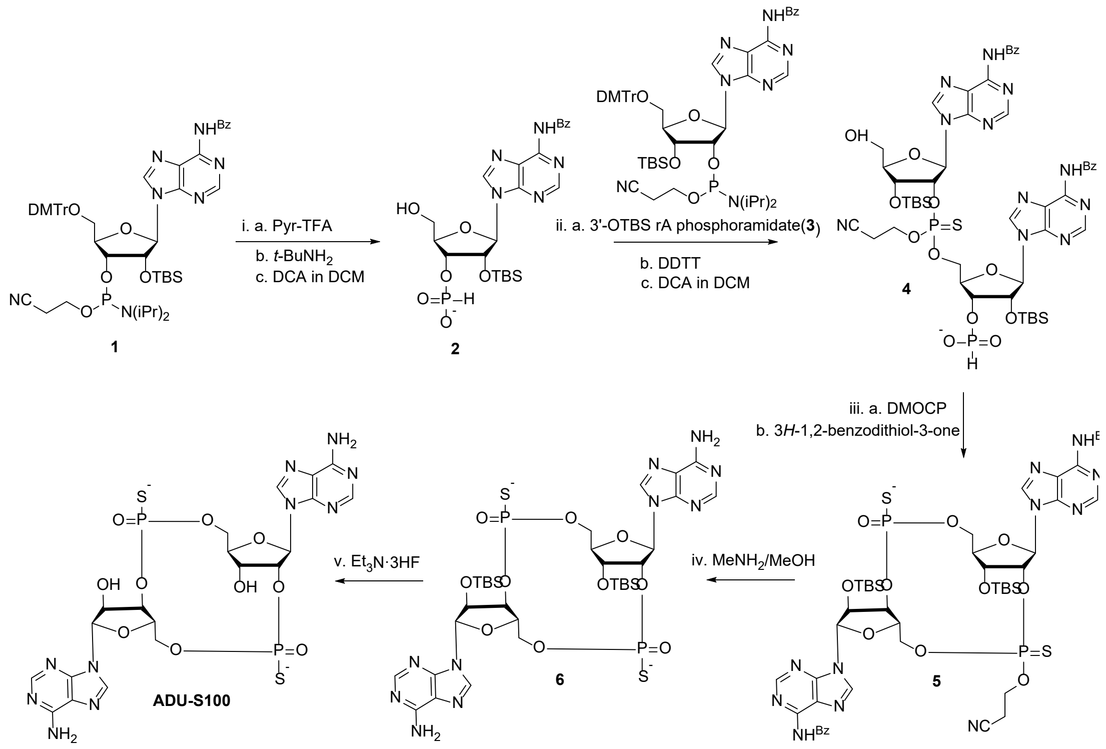

Supplementary Figure 1. Preparation of ADU-S100[1]**.** i) a. Pyr·TFA, CH_3CN, rt, 1 min; b. *t*-BuNH2, rt, 10 min; c. 3% DCA in DCM, 20 min; ii) a. 3'-OTBS rA phosphoramidite (**3**), CH_3CN, 5 min; b. DDTT, 30 min; c. 3% DCA in DCM, 20 min. iii) a. DMOCP, 30 min, rt, pyridine; b. 3*H*-1,2-benzodithiol-3-one, 20 min; iv) MeNH2 in MeOH, 30 °C, 3 h; v) Et_3N·3HF, 50 °C, 3h.

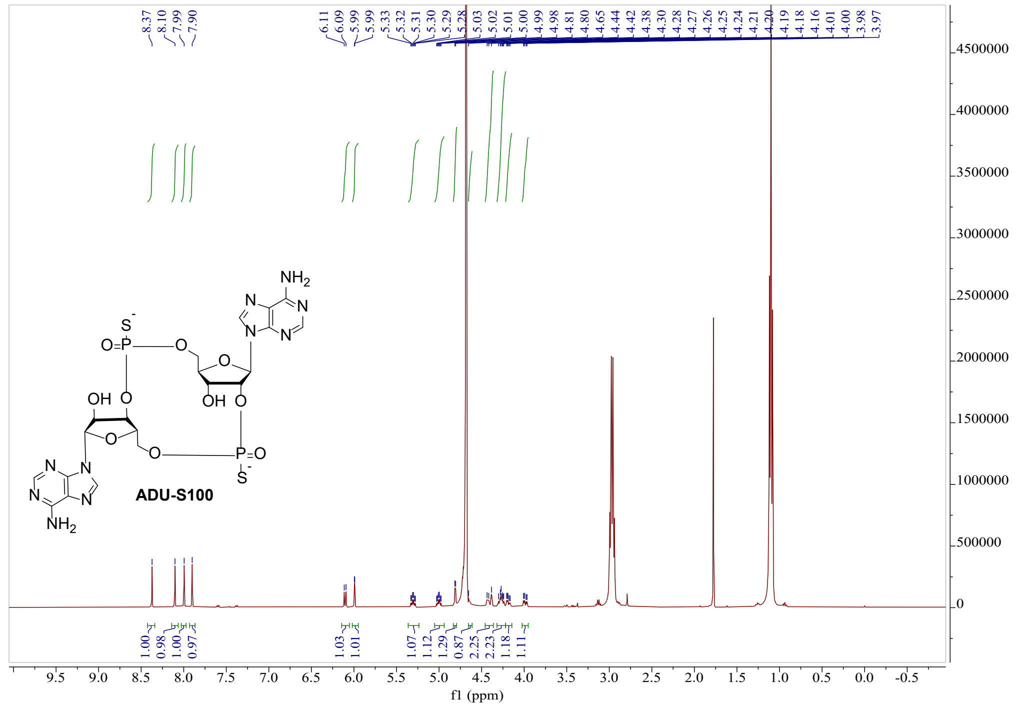

Supplementary Figure 2. ^{1}H NMR spectrum of ADU-S100

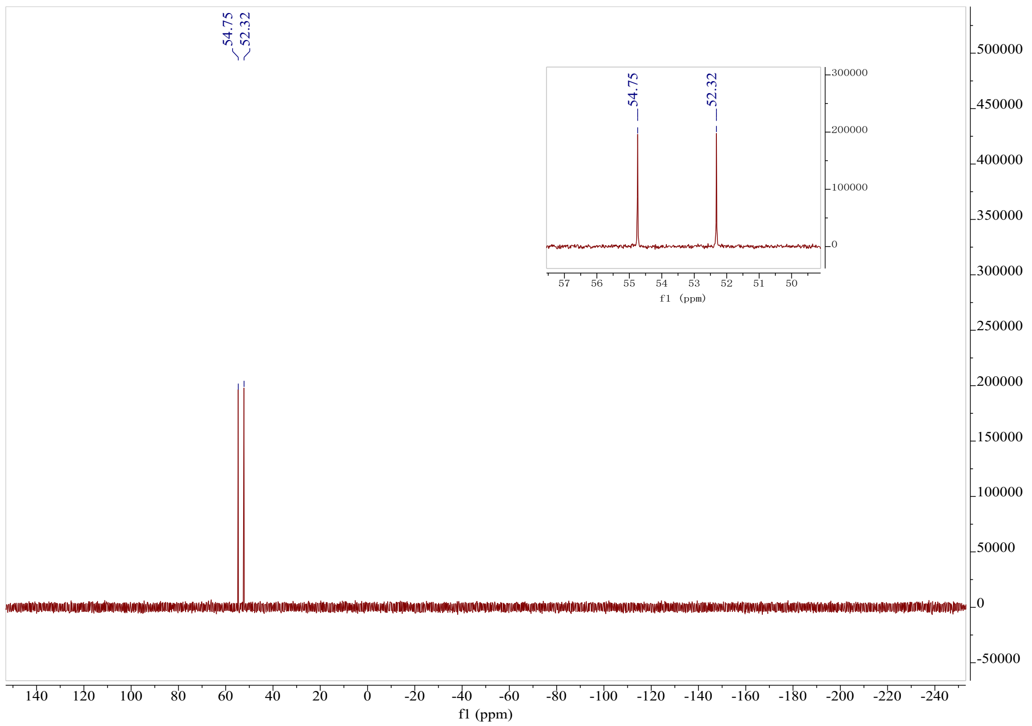

Supplementary Figure 3. ^{31}P NMR spectrum of ADU-S100

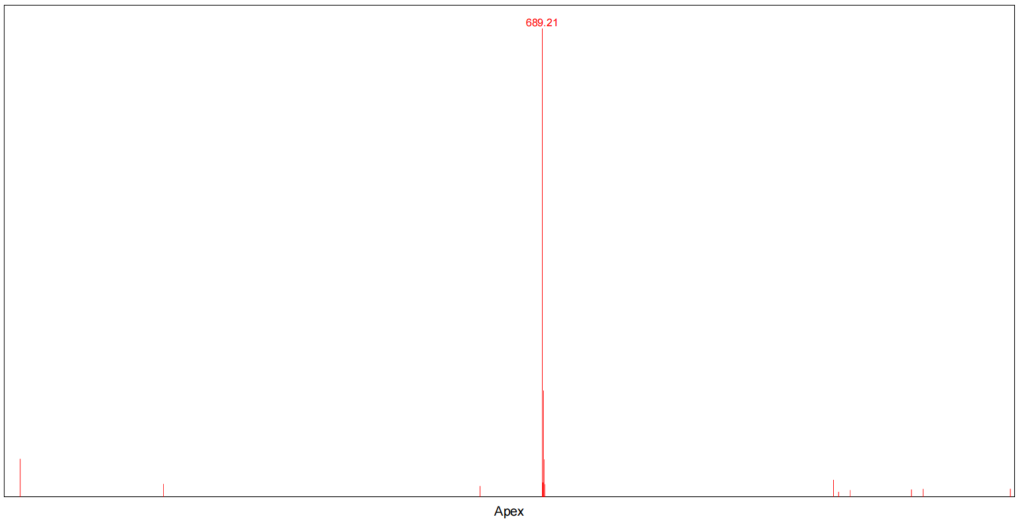

Supplementary Figure 4. MS spectrum of ADU-S100

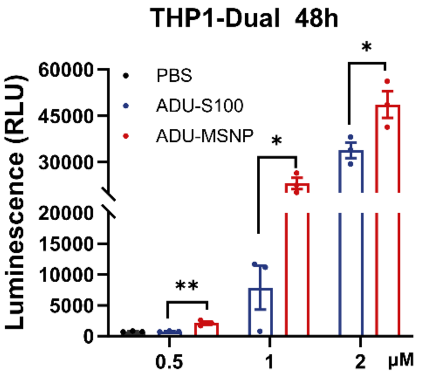

Supplementary Figure 5. Dose-dependent in THP1-Dual cells after 48 h treatment with ADU-S100 and ADU-MSNPs; RLU, relative light units (n=3). Data represent mean ± SEM. Statistical analysis was performed by two-tailed Student's *t*-test. *$p < 0.05$, **$p < 0.01$, ***$p < 0.001$.

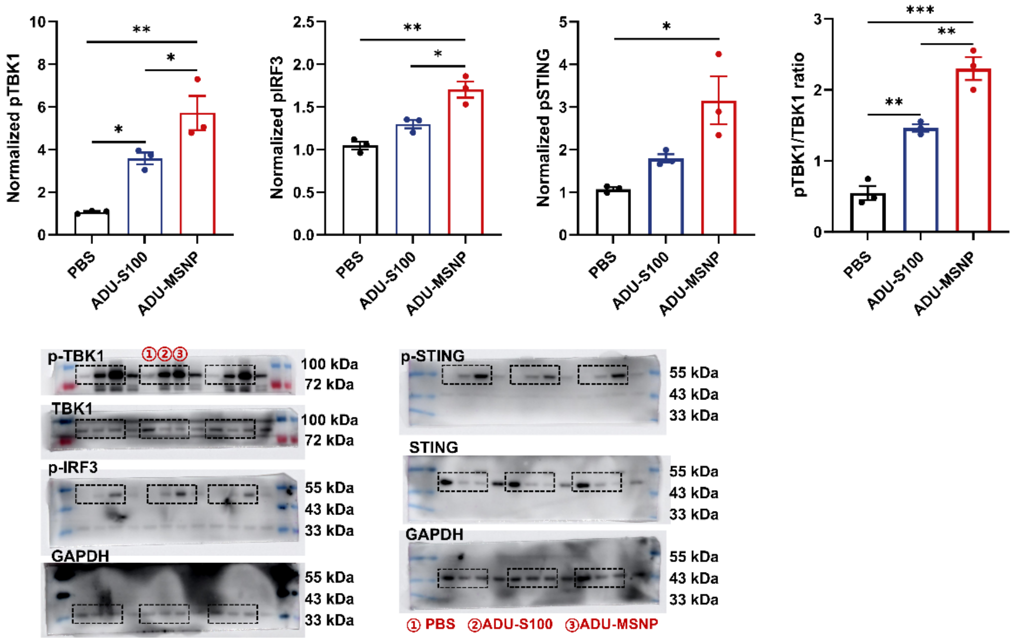

Supplementary Figure 6. Immunoblots densitometry of pTBK1, pIRF3, pSTING, and ratio of pTBK1 to TBK1 in BMDCs treated for 6 h with 1 μM ADU-S100 or ADU-MSNPs (n=3). Original scans of immunoblots in Figure 2D. Densitometry was quantified by ImageJ. Data represent mean ± SEM. Statistical analysis was performed by one-way ANOVA with Tukey's multiple comparisons test. *$p < 0.05$, **$p < 0.01$, ***$p < 0.001$.

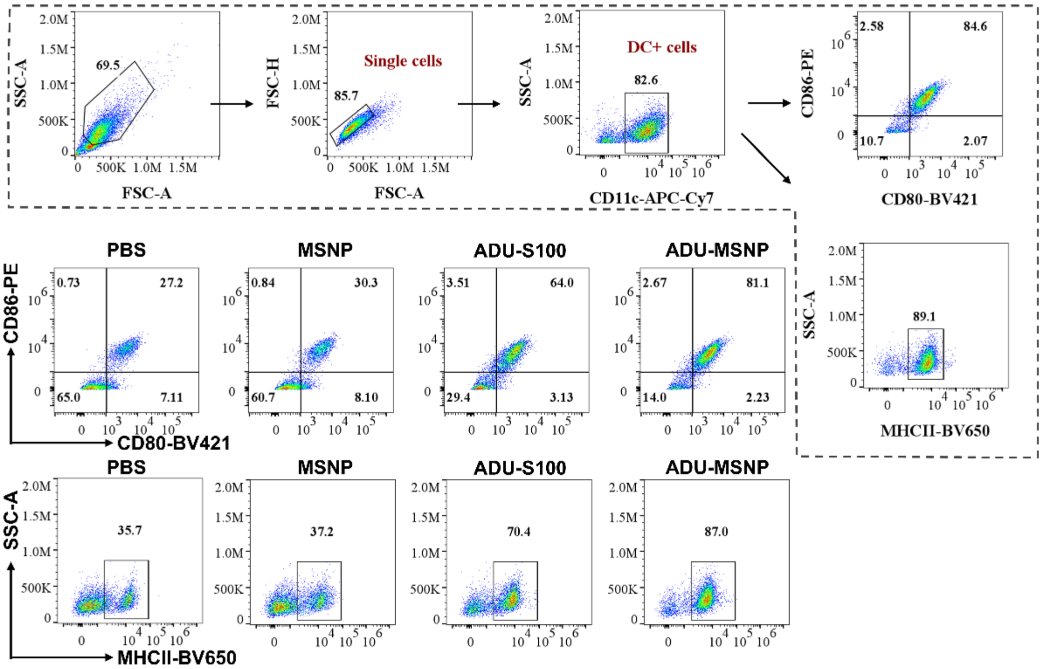

Supplementary Figure 7. Gating strategy for BMDC maturation and representative scatter plots related to Figure 2G-H.

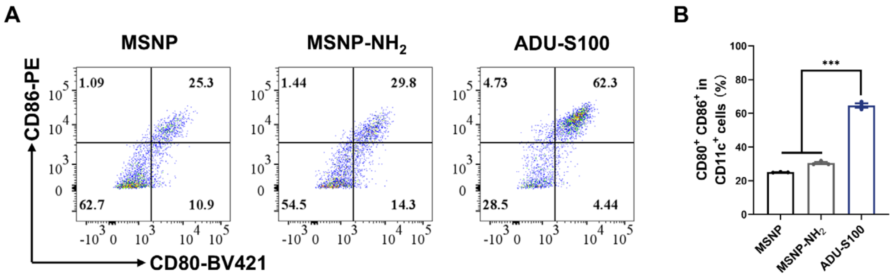

Supplementary Figure 8. Flow cytometry analysis of BMDC maturation following treatment with MSNP and MSNP-NH_2. (A-B) Representative scatter plots and quantification of combined CD80/CD86 expression. Data represent mean ± SEM. Statistical analysis was performed by one-way ANOVA with Tukey's multiple comparisons test. ***p < 0.001.

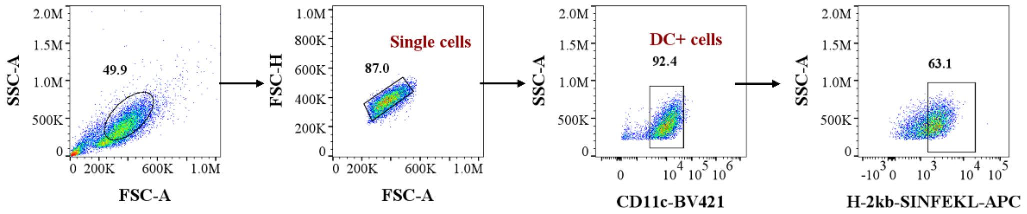

Supplementary Figure 9. Gating strategy for antigen cross-presentation in BMDCs related to Figure 2I.

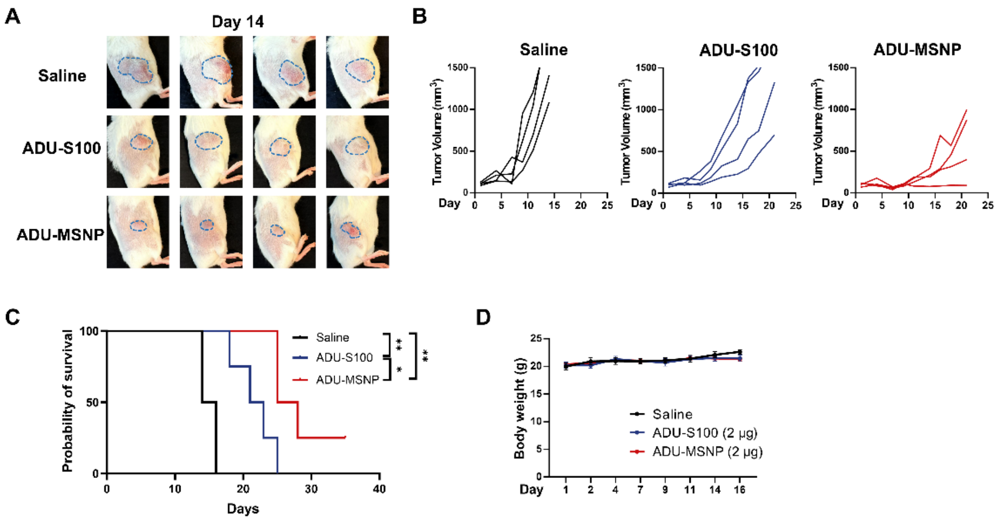

Supplementary Figure 10. Local intratumoral administration of ADU-MSNPs inhibits tumor progression. A) Representative tumor photographs on day 14 related to Figure 2N. B) Individual tumor growth curves of various treatments (n=4). C) Survival curves of tumor-bearing mice receiving the indicated treatments (n=4). D) Body weight of mice over the treatment period (n=4). Data represent mean ± SEM. Statistical analysis was performed by log-rank (Mantel-Cox) test. *$p < 0.05$, **$p < 0.01$, ***$p < 0.001$.

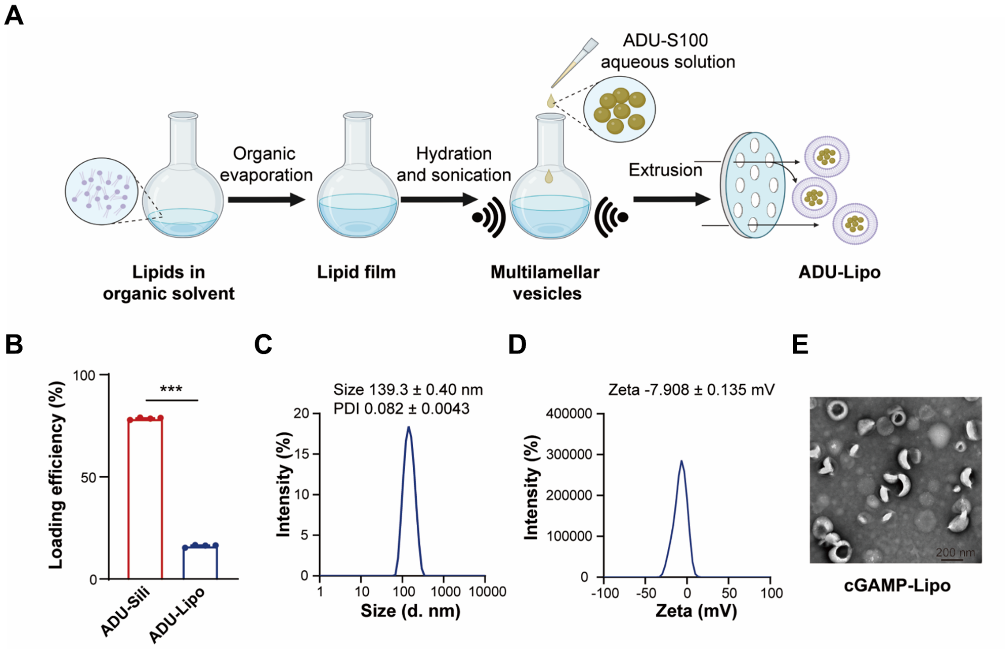

Supplementary Figure 11. Preparation and characterization of STING agonist-loaded liposomes. A) Synthesis route of CDN-Lipo. B) Loading efficiency of MSNPs and liposomes (n=4). C-D) Hydrodynamic diameter and zeta potential of cGAMP-Lipo (n=3). E) Representative TEM image of cGAMP-Lipo. Scale bar = 200 nm. Data represent mean ± SEM. Statistical analysis was performed by two-tailed Student's *t*-test. A was created using BioRender.

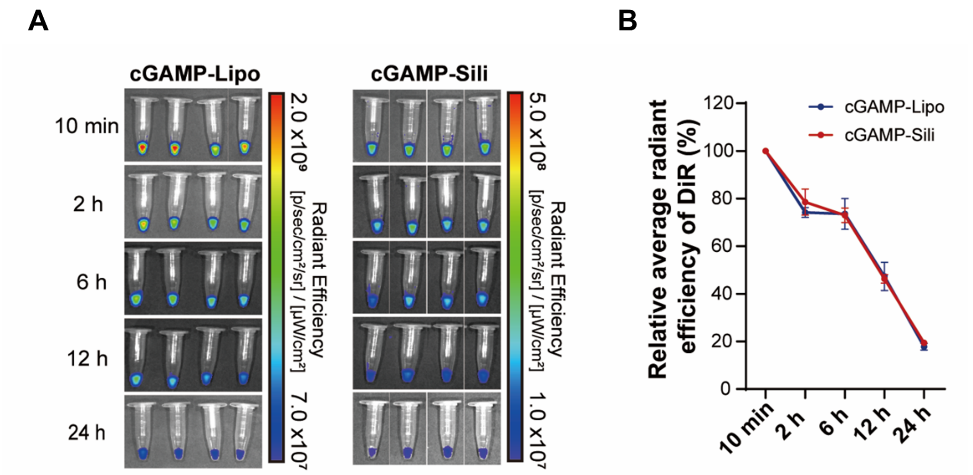

Supplementary Figure 12. A) Serum retention of DiR-labeled liposomes and silicasomes. Note: the absolute DiR intensity of the liposome sample is higher than that of the silicasome due to the lower cGAMP loading efficiency in liposomes. B) Quantification of relative average radiant efficiency of DiR (n=4). Data represent mean ± SEM.

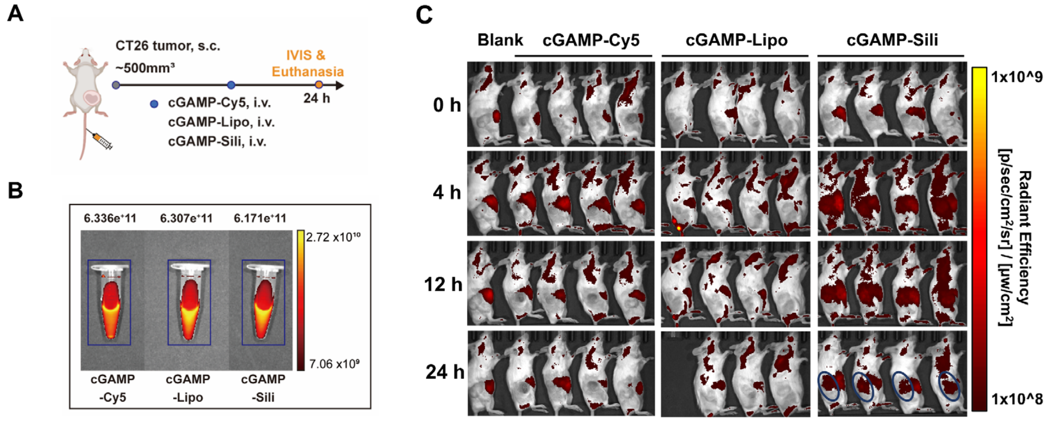

Supplementary Figure 13. A) CT26 tumor-bearing mice were intravenously injected with either free cGAMP-Cy5, cGAMP-Lipo, or cGAMP-Sili. Mice were euthanized and subjected to *in vivo* imaging of mice using IVIS. B) The fluorescence image of the injected sample, cGAMP-Cy5, cGAMP-Lipo, or cGAMP-Sili, showing similar mean fluorescence intensity of Cy5. C) Representative *in vivo* IVIS fluorescence images of administered mice (n=3-4). Note: one mouse in the liposome group died unexpectedly (unknown cause), leaving one fewer sample at 24 h.

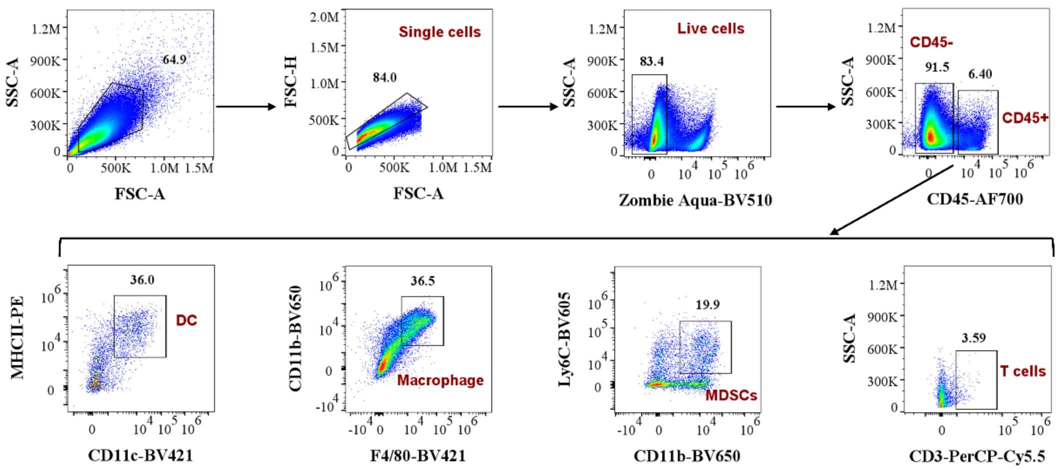

Supplementary Figure 14. Gating strategy for analyzing cellular internalization of cGAMP-Sili in TME related to Figure 3I.

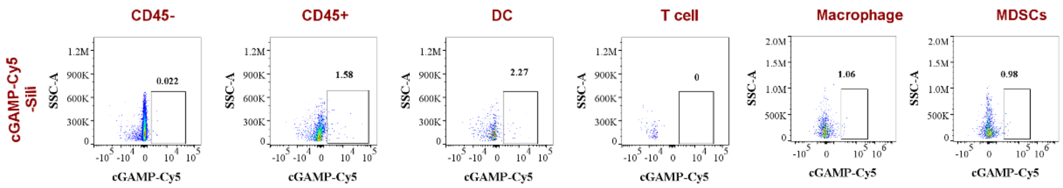

Supplementary Figure 15. Representative scatter plots of cGAMP-Sili uptake by various cell populations in TME related to Figure 3I.

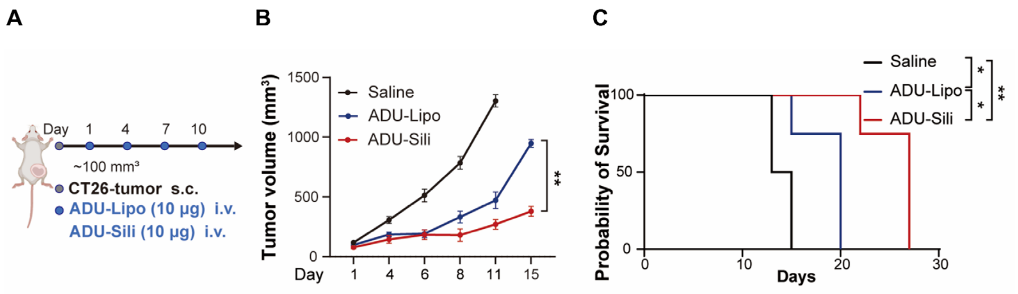

Supplementary Figure 16. Antitumor efficacy of systemically administered ADU-Lipo and ADU-Sili. A) Schematic timeline showing systemic administration of ADU-Lipo or ADU-Sili (containing 10 μg ADU-S100 per injection) on days 1, 4, 7, and 10 in CT26 tumor-bearing mice. (n=4). B) Average tumor growth curves of different groups (n=4). C) Survival curves of tumor-bearing mice receiving the indicated treatments (n=4). Data represent mean ± SEM. Statistical analysis was performed by two-way ANOVA with Dunnett's multiple comparisons test (B), or log-rank (Mantel-Cox) test (C). *$p < 0.05$, **$p < 0.01$, ***$p < 0.001$.

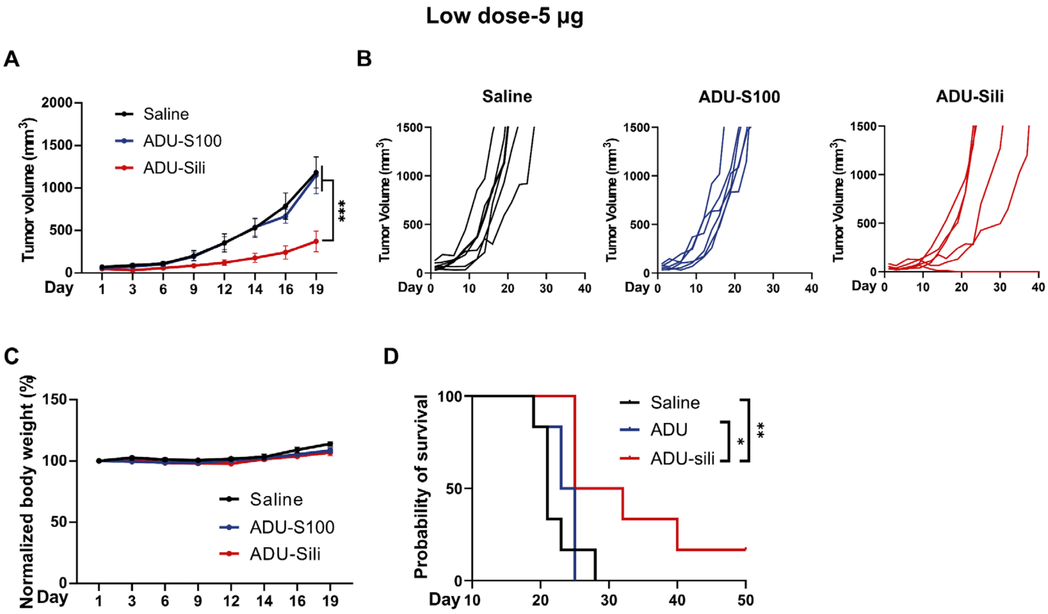

Supplementary Figure 17. Systemic intravenous administration of low-dose ADU-Sili exerts tumor suppression. A) CT26 subcutaneous tumor growth curves with ADU-S100 or ADU-Sili treatments, containing 5 μg ADU-S100 (n=6). B) Individual tumor growth curves of different groups (n=6). C) Body weight change over the treatment period (n=6). D) Survival curves of tumor-bearing mice receiving the indicated treatments (n=6). Data represent mean ± SEM. Statistical analysis was performed by two-way ANOVA with Tukey's multiple comparisons test (A), or log-rank (Mantel-Cox) test (C). *p < 0.05, **p < 0.01, ***p < 0.001.

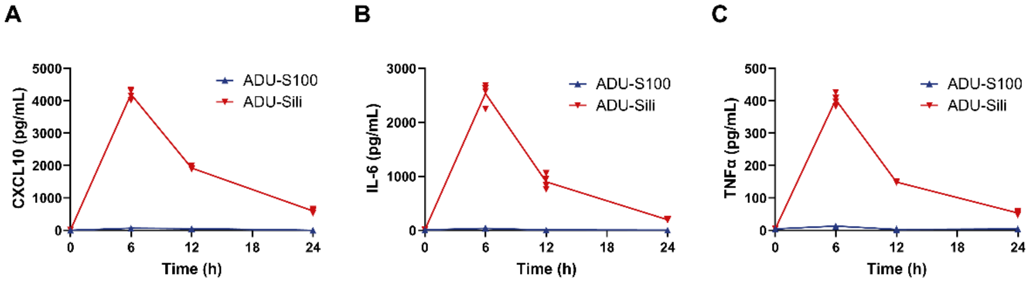

Supplementary Figure 18. Serum cytokine profiles are altered following systemic intravenous administration of ADU-Sili. Serum levels of CXCL10, IL-6, and TNF-α were measured 24 h after systemic intravenous administration of ADU-Sili (n=4). Data represent mean ± SEM.

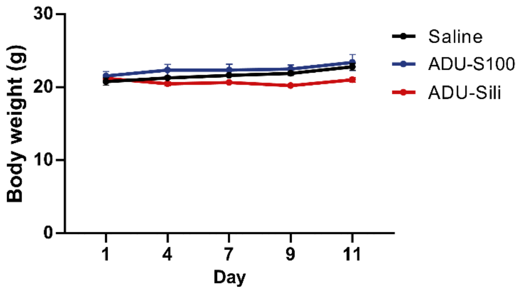

Supplementary Figure 19. Body weight of tumor-bearing mice over the treatment period related to Figure 4C (n=4). Data represent mean ± SEM.

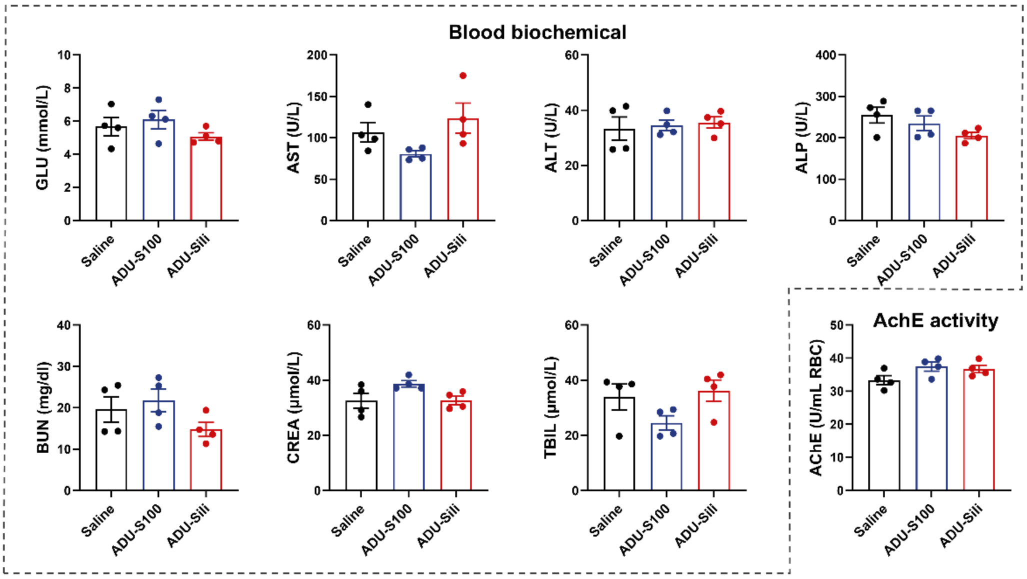

Supplementary Figure 20. Safety assessment of systemic intravenous administration of ADU-Sili. BALB/c mice were systemically injected with ADU-Sili (10 μg) on days 1, 4, 7, and 10. Serum biochemistry was measured 4 days post the final injection. Activity of acetylcholine esterase (AChE) in RBC was assessed at 2 h after the last injection (n=4). Data represent mean ± SEM.

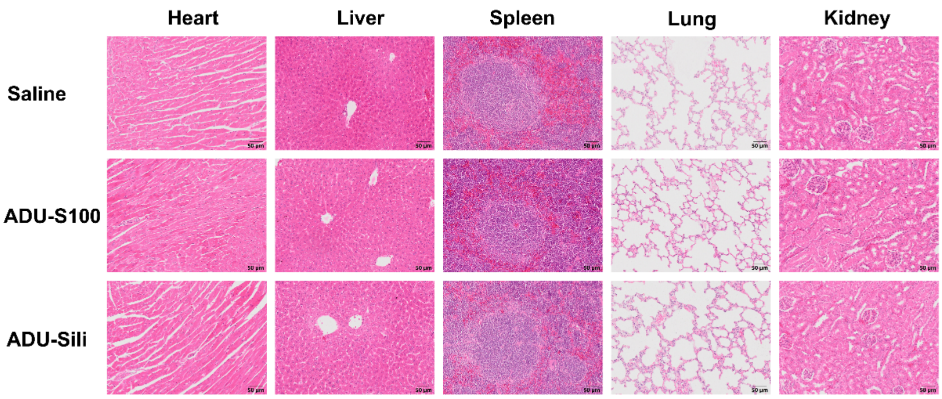

Supplementary Figure 21. Histological analysis of major organs after systemic administration of ADU-Sili. BALB/c mice were systemically injected with ADU-Sili (10 μg) on days 1, 4, 7, and 10. Organs were harvested for H&E staining at 4 days post the last injection (n=4). Scale bar = 50 μm.

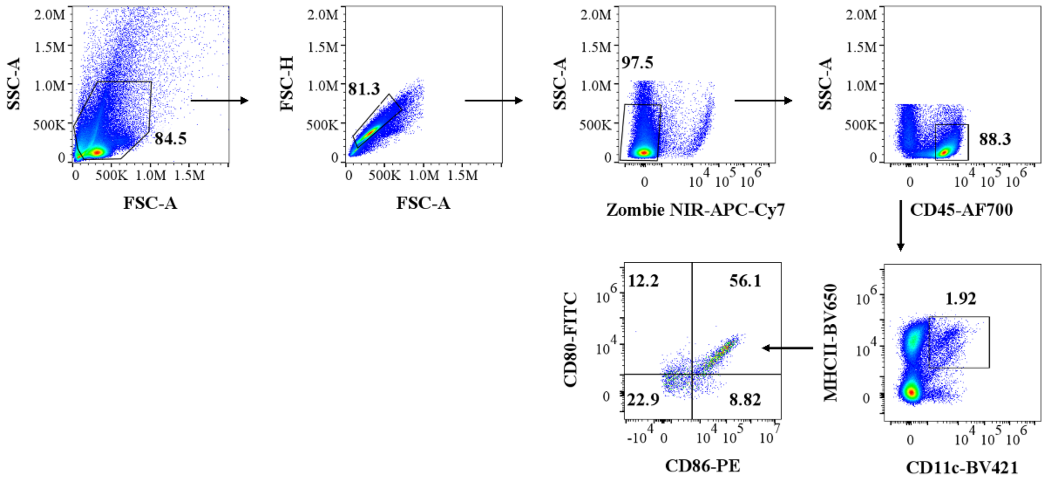

Supplementary Figure 22. Flow cytometry gating strategy for analyzing DC maturation in TDLNs after systemic administration of ADU-Sili, related to Figure 5B.

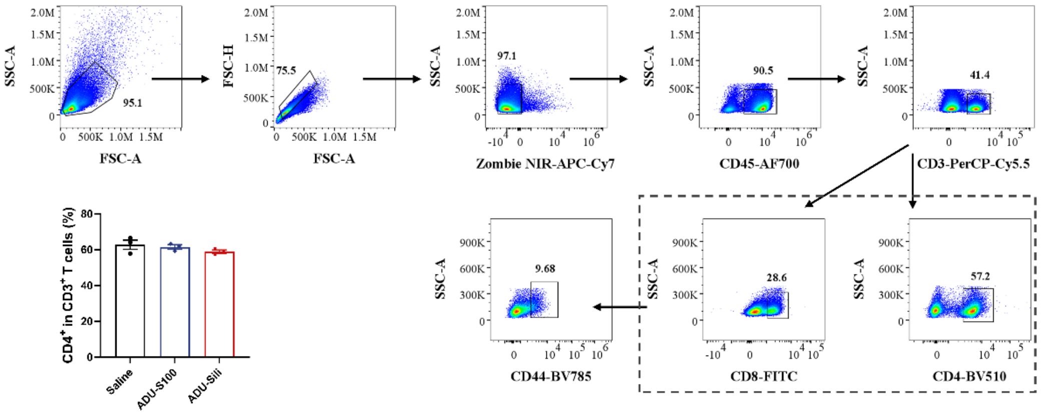

Supplementary Figure 23. Gating strategy for analyzing effector and memory T cells in spleen after systemic administration of ADU-Sili, related to Figure 5D-E. And the quantification of CD4⁺ T cells in the spleen (n=3). Data represent mean ± SEM. Statistical analysis was performed by one-way ANOVA with Tukey's multiple comparisons test. **p* < 0.05, ***p* < 0.01, ****p* < 0.001.

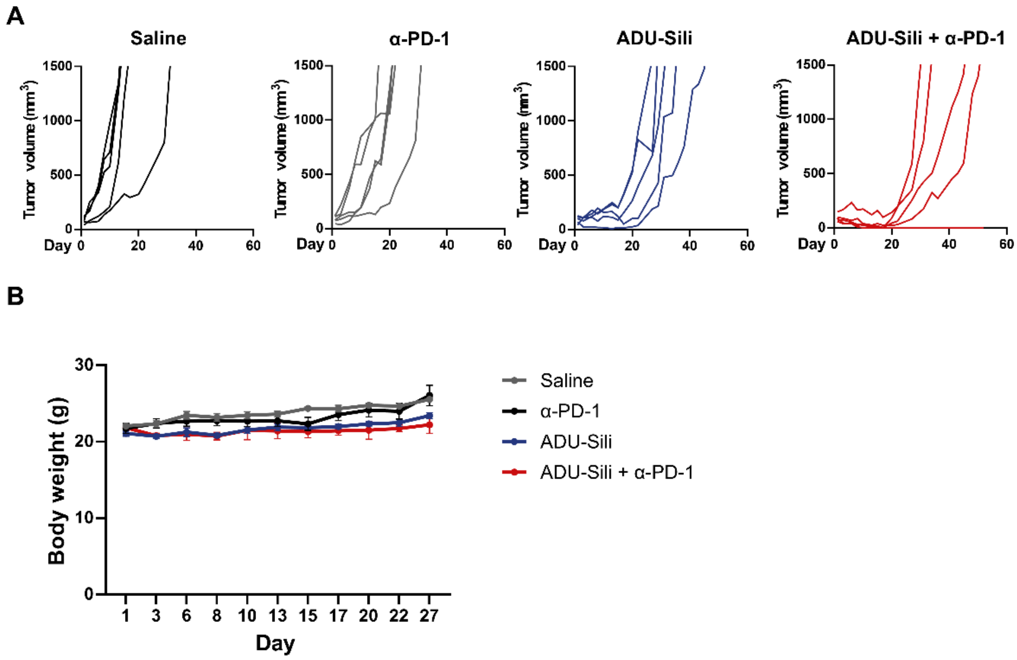

Supplementary Figure 24. A) Individual tumor growth curves for various treatments. B) Body weight of tumor-bearing mice over the treatment period (n=5). Data represent mean ± SEM.

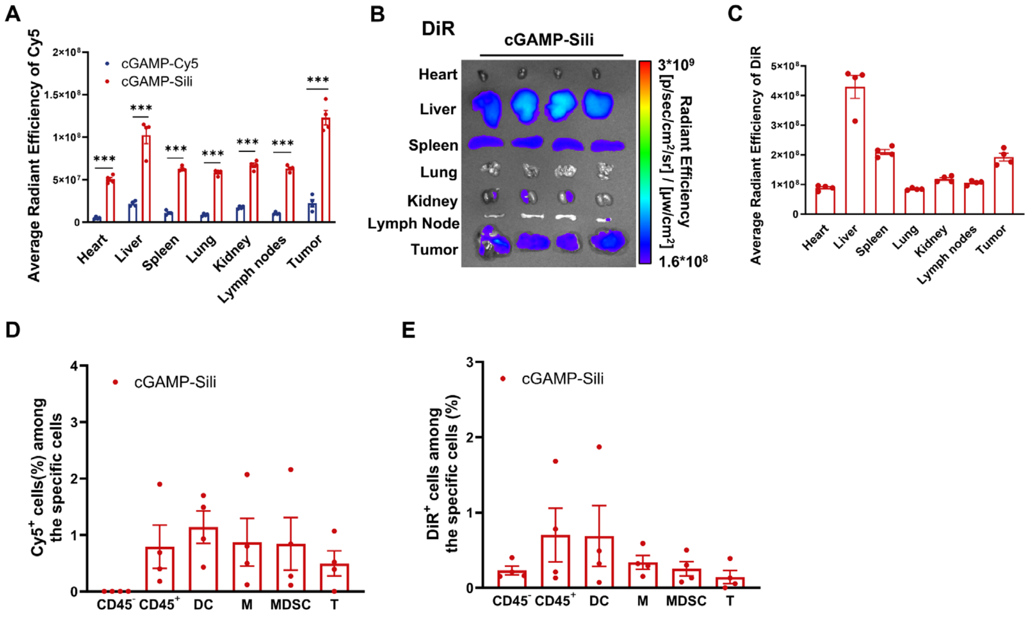

Supplementary Figure 25. Biodistribution of cGAMP-Sili after systemic administration for 24 h. A) Quantification of the average radiant efficiency of Cy5 across major organs related to Figure 7B (n=4). B-C) Biodistribution and quantification of DiR-labeled silicasomes in major organs (n=4). D) Flow cytometry analysis of cGAMP-Sili internalization among immune cell populations within the TME (n=4). E) Flow cytometry analysis of DiR-labeled silicasomes uptake among immune cell populations within the TME (n=4). Data represent mean ± SEM. Statistical analysis was performed by two-tailed Student's *t*-test (A, C-D). *$p < 0.05$, **$p < 0.01$, ***$p < 0.001$.

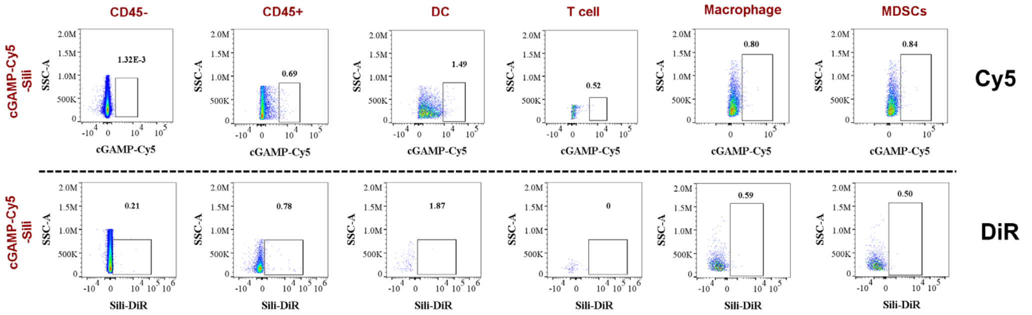

Supplementary Figure 26. Gating strategy for analyzing cellular uptake of cGAMP-Cy5 and DiR-labeled silicasomes in TME.

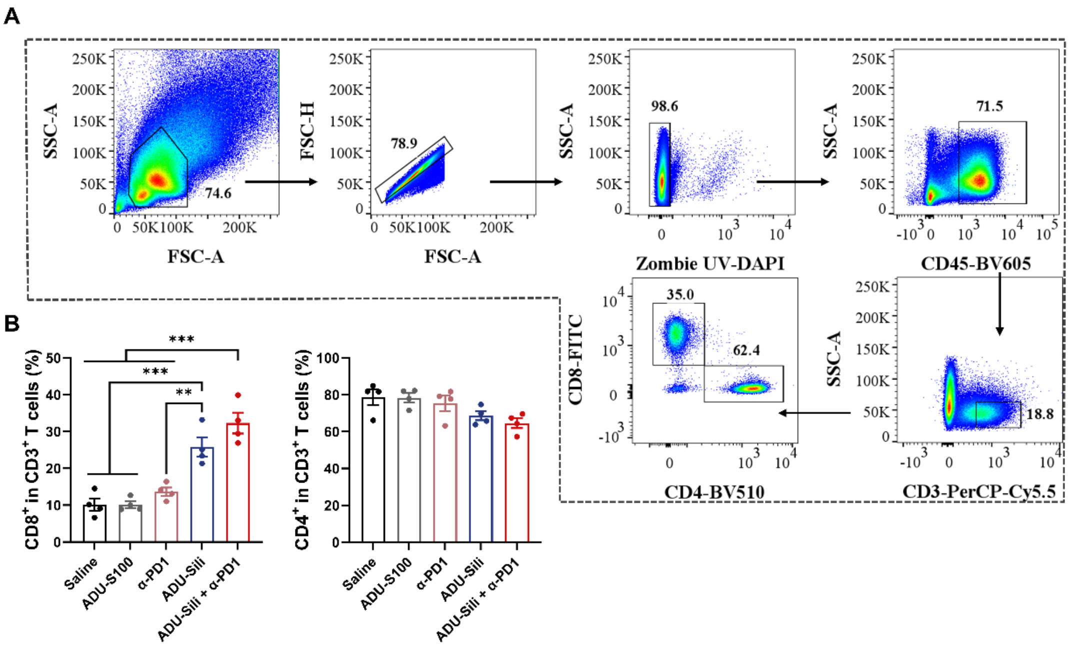

Supplementary Figure 27. Gating strategy for analyzing effector T cells in spleen after systemic administration, related to Figure 8A. The quantification of $CD8^+$ and $CD4^+$ T cells in the spleen (n=4). Data represent mean ± SEM. Statistical analysis was performed by one-way ANOVA with Tukey's multiple comparisons test. *$p < 0.05$, **$p < 0.01$, ***$p < 0.001$.

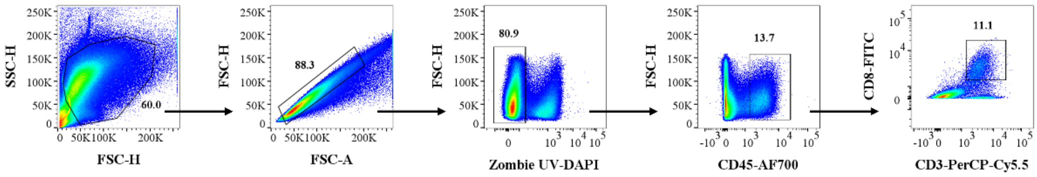

Supplementary Figure 28. Gating strategy for analyzing $CD8^+$ T cells in TME after systemic administration, related to Figure 8C.

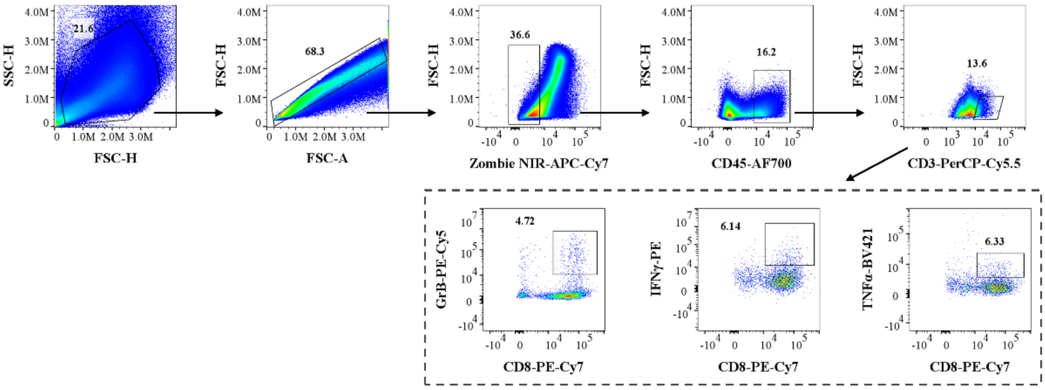

Supplementary Figure 29. Gating strategy for analyzing effector cytokines in $CD8^+$ T cells in TME after systemic administration, related to Figure 8E-J.

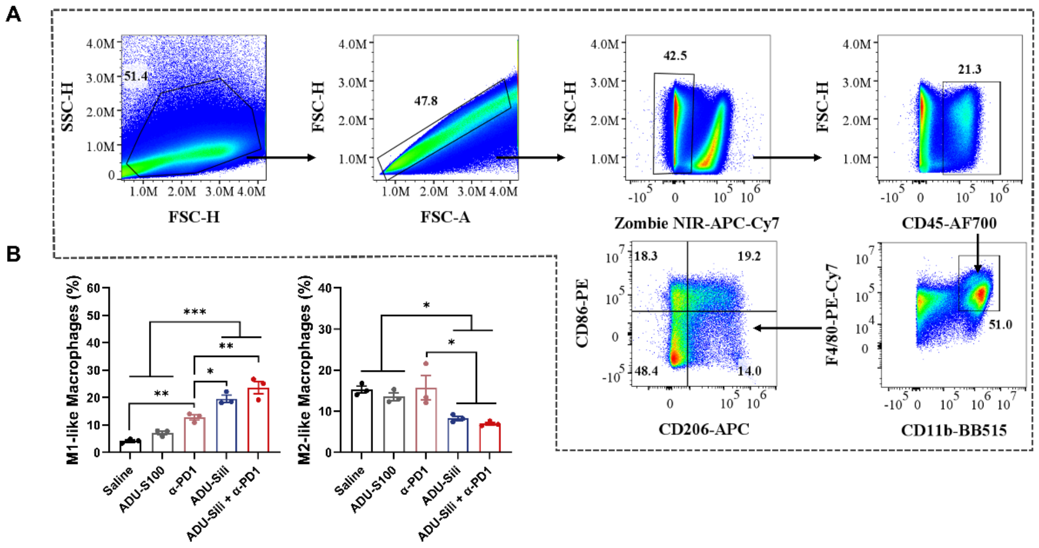

Supplementary Figure 30. Flow cytometry gating strategy for analysis of M1-like and M2-like macrophages in TME, related to Figure 8H. The quantification of M1-like and M2-like macrophages (n=3). Data represent mean ± SEM. Statistical analysis was performed by one-way ANOVA with Tukey's multiple comparisons test. **p* < 0.05, ***p* < 0.01, ****p* < 0.001.

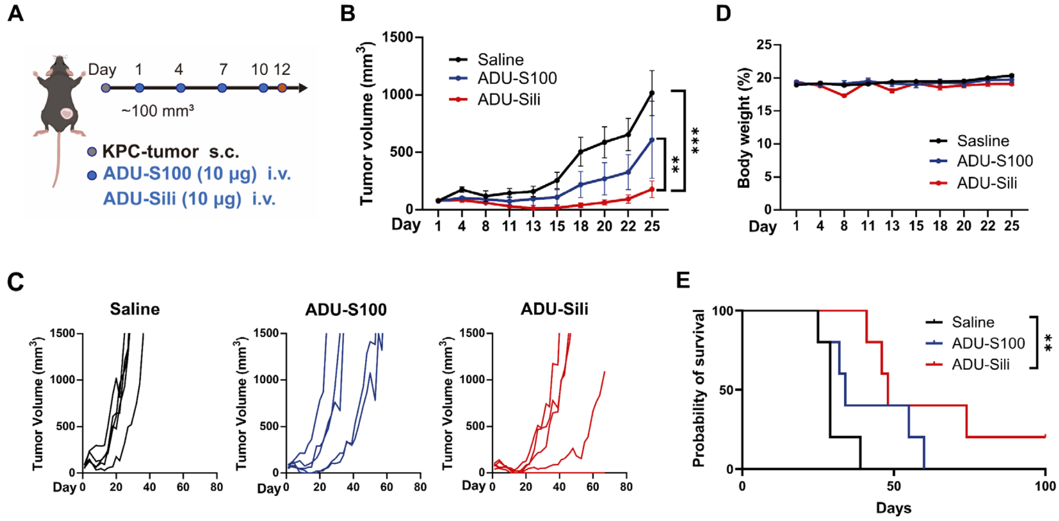

Supplementary Figure 31. Systemic administration of ADU-Sili exerts tumor suppression in the KPC tumor model. A) Schematic timeline showing systemic administration of ADU-S100 or ADU-Sili in KPC pancreatic tumor-bearing mice. B) Average tumor growth curves of different groups (n=5). C) Individual tumor growth curves of different groups (n=5). D) Body weight change over the treatment period (n=5). E) Survival curves of tumor-bearing mice receiving the indicated treatments (n=5). Data represent mean ± SEM. Statistical analysis was performed by two-way ANOVA with Tukey's multiple comparisons test (B), or log-rank (Mantel-Cox) test (E). **p* < 0.05, ***p* < 0.01, ****p* < 0.001.

Reference

[1] Gaffney B L, Veliath E, Zhao J, Jones Roger A. One-flask syntheses of c-di-GMP and the [Rp,Rp] and [Rp,Sp] thiophosphate analogues. Org Lett, 2010, 12(14): 3269-3271. https://doi.org/10.1021/ol101236b.
